# Expression of myelin transcription factor 1 and lamin B receptor mediate neural progenitor fate transition in the zebrafish spinal cord pMN domain

**DOI:** 10.1016/j.jbc.2022.102452

**Published:** 2022-09-05

**Authors:** Lingyan Xing, Rui Chai, Jiaqi Wang, Jiaqi Lin, Hanyang Li, Yueqi Wang, Biqin Lai, Junjie Sun, Gang Chen

**Affiliations:** 1 Key Laboratory of Neuroregeneration of Jiangsu and the Ministry of Education, Co-Innovation Center of Neuroregeneration, NMPA Key Laboratory for Research and Evaluation of Tissue Engineering Technology Products, Nantong University, Nantong, China; 2 Department of Physiology, School of Medicine, Nantong University, Nantong, China; 3 School of Medicine, University of Utah, Salt Lake City, Utah, USA; 4 Key Laboratory for Stem Cells and Tissue Engineering (Sun Yat-sen University), Ministry of Education, Co-Innovation Center of Neuroregeneration, Nantong University, Nantong, China; 5 Basic Medical Research Center, School of Medicine, Nantong University, Nantong, China

**Keywords:** single-cell RNA sequencing, zebrafish, oligodendrocyte progenitor cells, radial glia, interneurons, motor neurons, pMN progenitor cells, CaP, caudal primary, CDNAs, complementary DNAs, CNS, central nervous system, DEG, differentially expressed gene, FACS, fluorescence activated cell sorting, hpf, hour post fertilization, MiP, middle primary, OPC, oligodendrocyte progenitor cell, PAGA, partition-based graph abstraction, pri-MN, primitive motor neuron, pri-OPC, primitive oligodendrocyte progenitor cell, RoP, rostral primary, scRNA-seq, single-cell RNA sequencing, UMAP, Uniform Manifold Approximation and Projection

## Abstract

The pMN domain is a restricted domain in the ventral spinal cord, defined by the expression of the *olig2* gene. Though it is known that the pMN progenitor cells can sequentially generate motor neurons and oligodendrocytes, the lineages of these progenitors are controversial and how their progeny are generated is not well understood. Using single-cell RNA sequencing, here, we identified a previously unknown heterogeneity among pMN progenitors with distinct fates and molecular signatures in zebrafish. Notably, we characterized two distinct motor neuron lineages using bioinformatic analysis. We then went on to investigate specific molecular programs that regulate neural progenitor fate transition. We validated experimentally that expression of the transcription factor *myt1* (myelin transcription factor 1) and inner nuclear membrane integral proteins *lbr* (lamin B receptor) were critical for the development of motor neurons and neural progenitor maintenance, respectively. We anticipate that the transcriptome features and molecular programs identified in zebrafish pMN progenitors will not only provide an in-depth understanding of previous findings regarding the lineage analysis of oligodendrocyte progenitor cells and motor neurons but will also help in further understanding of the molecular programming involved in neural progenitor fate transition.

A detailed analysis of the types of cells in the developing central nervous system (CNS), especially neural stem cells/progenitors, will reveal how these cells are generated, assist in elucidating the pathology of neurodevelopmental disorders, and suggest ways to direct them to target specific cell types for disease treatment. In development, neural progenitors have potential to generate neurons and glia, which is the basis of complexity in the CNS. In the spinal cord, both motor neurons and oligodendrocytes arise from the pMN domain; progenitors in this domain are defined by the gene marker *olig2* and sequentially form motor neurons and oligodendrocyte progenitor cells (OPCs). This process is highly regulated by a series of transcription factors. Neurogenin1 and Neurogenin2 are upregulated preceding neurogenesis but downregulated in oligodendrogenesis (1). Olig2 and Nkx2.2 together promote generation of the OPC lineage (2).

Though multiple studies have revealed several key factors in the development of motor neurons and OPCs, the fates and transcriptional signatures of progenitors are not fully understood. In addition, the developmental lineages of motor neurons and OPCs are controversial (3). For example, individual progenitors grown in culture or traced by recombinant retroviral infection or fluorescent dye can generate both neurons and glia in flies, mice, or zebrafish (4, 5, 6, 7). However, clonal lineage testing and live imaging reveal that subsets of progenitors are fate-restricted, forming either motor neurons or glial cells in mice or zebrafish (3, 8). In addition, though not as commonly mentioned as motor neurons and oligodendrocytes, astrocytes and interneurons are also observed in the clones from the pMN progenitors (7, 9). These findings from tissue transplantation, lineage tracing, or cell ablation have been heavily based on reporter genes, the non-specificity of which makes it difficult to generate a model that fits the data, resulting in incomplete characterization of molecular regulators. The single-cell RNA sequencing (scRNA-seq) approach offers a high resolution method to decipher the molecular programs driving the transition of neural fates. Recent studies have used large-scale profiling of single cells in zebrafish (10, 11), but these studies focus on early embryogenesis, before most glial cells are specified.

Here, we applied the scRNA-seq technique to sequence 24,646 cells in zebrafish ventral spinal cords at 28 hpf (hour post fertilization), 42 hpf, and 60 hpf, identify temporal lineages, and decode the transcriptome landscapes for progenitor fate transitions. Our study reveals the heterogeneity of *olig*^2+^ progenitor cells, which not only include the known pMN progenitors for the motor neuron and OPC lineages but also two distinct neural progenitor populations for interneurons and a second lineage of motor neurons. We also deciphered critical molecules for neural progenitor fate transitions and demonstrate that *myt1* (Myelin Transcription Factor 1) is necessary for motor neuron development and that *lbr* (lamin B receptor) is necessary for the maintenance of neural progenitor cell stemness. Interestingly, the myt1 mutant does not lead to a deficiency in the OPC lineage, indicating that signals from early formed motor neurons are not obligatory for the development of the OPC lineage. *Lbr* is important for the maintenance of neural stem/progenitor cell stemness. Our study unveils molecular signatures of pMN progenitors, deciphers their novel molecular programming, and suggests approaches for multiple applications, including stem cell transplantation as well as directed cell differentiation or reprogramming. Notably, we also have established open web-based datasets for researchers to access.

## Results

### A single-cell atlas of pMN progenitors and their progeny

**Figure 1:**
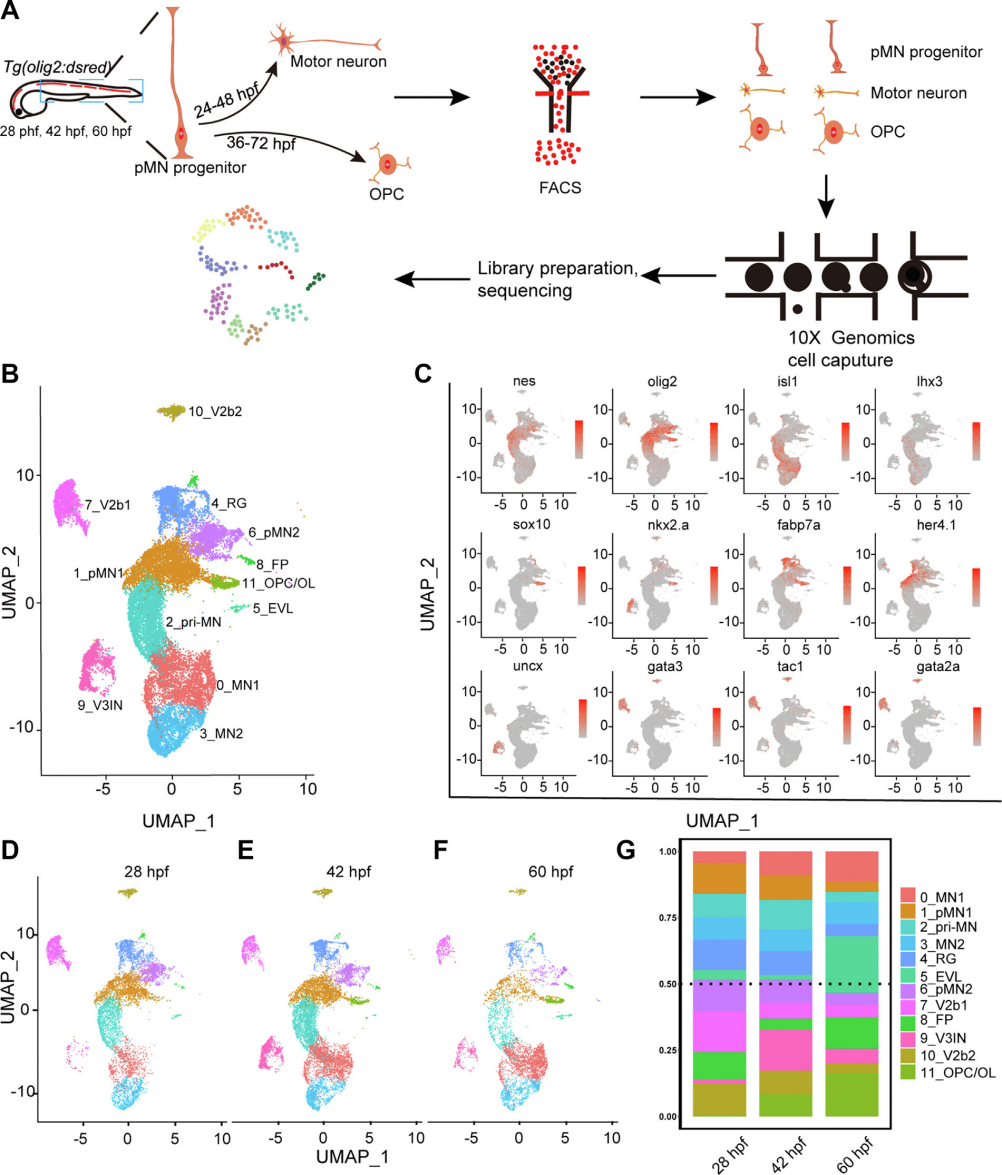
**Single-cell transcriptome maps of *olig*2**^**+**^**cells in the zebrafish spinal cords.***A*, diagrams of the scRNA-seq workflow. *B*, UMAP clustering of single-cell samples. *Colors* indicate distinct groups. *C*, the expression of marker genes visualized by UMAP. The *colors* denote the expression values. The darker the color, the higher the expression. *D*–*F*, cell subpopulations across three different developmental time points at 28 hpf, 42 hpf, and 60 hpf. *G*, percentages of cell types at different time points. EVL, enveloping layer; FP, floor plates; hpf, hour post fertilization; pMN, pMN progenitors; RG, radial glia; scRNA-seq, single-cell RNA sequencing; UMAP, Uniform Manifold Approximation and Projection; V2b, V2b interneurons; V3IN, V3 interneurons.

Progenitors in the ventral spinal cords (pMN domains) of zebrafish initiate differentiation into motor neurons prior to 24 hpf, and motor neuron generation is largely accomplished by 48 to 51 hpf (12). At 36 hpf, *sox10* and *nkx2.2* positive cells (OPC lineage cells) have been observed (13). In this transgenic *Tg(olig2:dsRed)*, the *olig2* regulatory element controls the expression of dsRed, which labels progenitors expressing *olig2* and their progeny with transient preservation of fluorescent protein from their progenitors (14). To identify the transcriptome profiles in pMN progenitors and their offspring, we applied fluorescence activated cell sorting (FACS) to specifically sort cells labeled by the transgenic line *Tg(olig2:dsRed)* at 28 hpf, 42 hpf, and 60 hpf, stages when active motor neuron development, OPC specification, and differentiation were occurring. To specifically identify progenitors and their offspring in the spinal cords, we only retained the fish trunks for single cell isolation (Fig. 1*A*). Approximately, 200 fish trunks at each time point were collected to minimize individual variation. Altogether, we transcriptionally profiled 24,646 high-quality cells with the high-throughput single-cell system 10× Genomics (15). A median of 4912 UMI counts and 1646 genes were detected (Fig. S1, *A*–*D*). This depth is comparable to similar studies (15). Sorted cells were classified based on their gene expression pattern. RNA-seq expression patterns were projected into UMAP (Uniform Manifold Approximation and Projection), a nonlinear dimensionality reduction algorithm, for cell population annotation (16). Altogether, 12 distinct cell clusters were identified (Fig. 1*B*).

By combining known gene markers and transcriptionally unique signatures (Fig. 1*C* and Supplemental Data 1), we defined these cells: pMN progenitors, motor neurons, radial glia, OPC, and interneurons. pMN progenitors were defined by their expression of *olig2* and *nestin* (7). Motor neurons were enriched for *mnx2a*, *mnx2b*, *mnx1*, *lhx3*, or *isl1* (17). Radial glia were defined by *fabp7* and *gfap* (18). OPC was enriched in *sox10*, *nkx2.2a*, and *prdm8* (15). Finally, V2b interneurons were marked by *gata3*, *tal1*, and *gata2a*, and V3 interneurons by *uncx* (19, 20, 21) (Figs. 1, *B* and *C* and S2, *A*–*L*, Supplemental Data 1). These results were in line with the cell types labeled by this transgenic line in previous work (22), confirming the reliability of our sequencing data.

Cell clustering analysis at 28 hpf, 42 hpf, and 60 hpf revealed an overlap between most annotated cells (Fig. 1, *D*–*F*). For example, clusters pMN_1 and pMN_2 exist in all three time points. However, the percentages of cell components changed dramatically (Fig. 1*G*). The fractions of motor neuron_1 significantly increased from 28 hpf to 60 hpf. OPCs were barely observable at 28 hpf, but greatly expanded at 42 hpf, and accumulated even more by 60 hpf. In contrast, pMN_1 and pMN_2 gradually decreased with time. These data indicate a rapid transition to differentiation.

**Figure 2:**
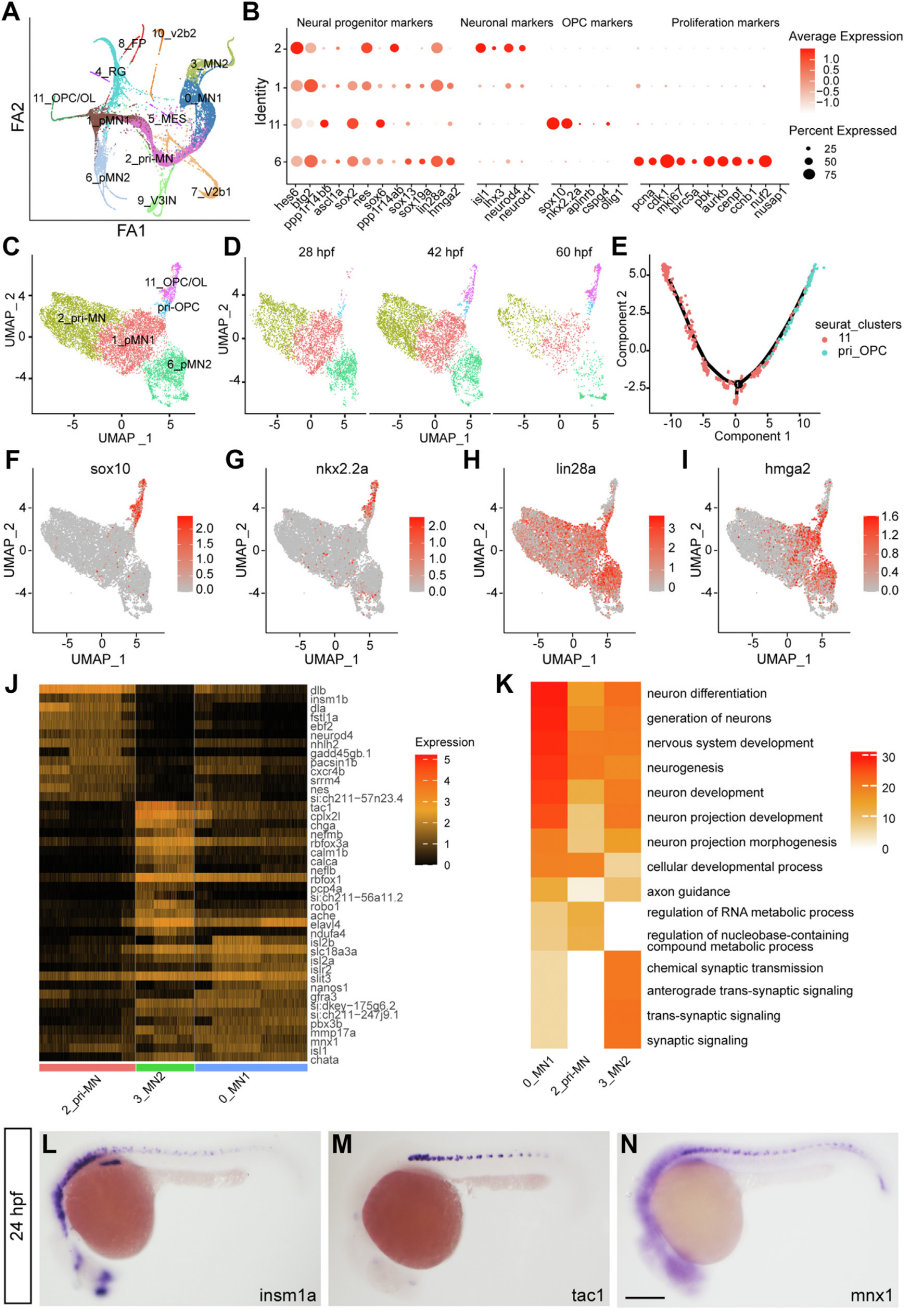
**Heterogeneity and lineage inference analyses of pMN progenitors for motor neurons and OPCs.***A*, cell trajectory analysis by Partition-based Graph Abstraction (PAGA). *B*, dot plot reveals expression levels of marker genes for progenitors, neurons, OPCs, and proliferating cells. Dot size represents the percentage of marker genes expressed in each cell population, and the color depicts the average expression of each gene. *C* and *D*, UMAP of pri-OPCs. *E*, lineage inference analysis by Monocle in the pri-OPCs and OPCs. *F*–*I*, UMAP of markers in the OPC (*F* and *G*) and progenitors (*H* and *I*) reveals the existence of pri-OPCs. *J* and *K*, enriched markers (*J*) and Go terms (*K*) in the pri-MN, MN_1, and MN_2. *Color scales* denote expression levels (*J*) and the statistical significance (*K*), respectively. *L*–*N*, *in situ* hybridization confirms RNA patterns of the pri-MN marker *insm1a* and MN_2 marker *tac1*, which have similar expression pattern as the neuronal marker mnx1 in the spinal cord. The scale bar represents 100 μm. OPC, oligodendrocyte progenitor cell; pri-MN, primitive motor neuron; pri-OPC, primitive oligodendrocyte progenitor cell; UMAP, Uniform Manifold Approximation and Projection.

To probe the potential relationships between the cells isolated by FACS, we performed partition-based graph abstraction (PAGA) analysis (23), which showed the topography and lineage of the cell clusters (Fig. 2*A*). The motor neurons and OPCs branched from the pMN progenitors. To better understand the transition, we characterized the cellular heterogeneity and features of the progenitors involved in these two lineages (including Clusters 0, 1, 2, 3, 6, 11). Markers including *nes (nestin)*, *hes6*, *and btg2* were used to identify progenitors, in which four populations (Clusters 1, 2, 6, 11) stood out (Fig. 2*B*). Proliferation markers, such as *pcna*, *cdk1*, and *mki67*, were all highly enriched in Cluster 6, indicating these were cycling progenitors (Fig. 2*B*). Cluster 2 was expressed with neuronal markers, such as *isl1* and *neurod4*, as well as multiple progenitor markers, including *nes* and *hes6*, indicating these were primitive motor neurons (pri-MNs). A subpopulation in 11 was denoted as OPCs, as it was enriched for *sox10* and *nkx2.2a* (Fig. 2*B*). Interestingly, progenitor markers, including *hes6*, *btg2*, *ppp1r14bb*, *ascl1a*, *sox2*, *nes*, *sox6*, *ppp1r14ab*, and *sox13*, were differentially expressed in the four populations (Clusters 1, 2, 6, and 11). *btg2* was enriched in pMN_1 but was significantly decreased in the pri-MNs and OPCs; *hes6* and *nes* had the highest expression in the pri-MNs; *ppp1r14bb*, *sox2*, and *sox6* were mainly enriched in the OPCs, but *ascl1a* and *nes* were barely observable in this population (Fig. 2*B*).

In the cell cluster OPC/OL, we observed some cells expressing *mbpb*, a mature oligodendrocyte marker, indicating that this cluster was a mixture of OPCs and mature oligodendrocytes (Fig. S2*A*). Trajectory inference analysis revealed the nature of the transition from OPCs to oligodendrocytes (Fig. S2, *B*–*D*). Consistently, genes in this subcluster with *mbpb* expression were enriched in myelination and cytoskeleton organization (Fig. S2*E*).

Go term analysis of marker genes reveals the developmental features in each subpopulation: for example, Cluster_1 (pMN_1) was enriched in tissue development and nervous system development. Cluster_2 (pri-MN) was enriched in nervous system development and neuron fate specification; Cluster_6 (pMN_2) was enriched in the cell cycle. Consistently, they were in concert with cell structural and morphological alterations. For example, Cluster_1 (pMN_1) was enriched in the anatomical structure formation involved in morphogenesis, Cluster 2 (pri-MN) was enriched in the cell projection assembly, and Cluster_11 (OPC) was enriched in the actin cytoskeleton organization (Fig. S3, *A*–*D*). Notably, highly expressed genes in each population were also enriched in distinct metabolic pathways. For example, Cluster 1 (pMN_1) was enriched in the regulation of biosynthetic processes, Cluster 2 (pri-MN) was enriched in the regulation of nucleobase-containing compound metabolic processes, Cluster 6 (pMN_2) was enriched in the regulation of nitrogen compound metabolic processes and the hydrogen peroxide catabolic processes, and Cluster 11 (OPC) was enriched in cellular component biogenesis and also in the generation of precursor metabolites and energy (Fig. S3, *A*–*D*). This evidence supports the massive energy and biosynthetic demands required for the cell cycle and differentiation. These four clusters can also be clearly defined by the genes involved in metabolic pathways and morphological alterations (Supplemental Data 3 and Fig. S3, *E* and *F*). Therefore, pMN progenitors or precursors for the motor neuron and OPC lineages were featured with distinct progenitor markers, developmental pathways, and metabolic pathways.

Interestingly, the UMAP plot reveals a small subpopulation in pMN_1 (Fig. 2*C*), which was enriched in progenitor markers, including *lin28a* and *hmgn2*, but with little expression of *nkx2.2a* and *sox10* (Fig. 2, *F*–*I*). This indicates that these are likely pri-OPCs (primitive OPCs). Consistently, pseudotime analysis revealed the developmental trajectory from pri-OPCs to OPCs (Fig. 2*E*). This subpopulation was observable at 28 hpf and still retained at 60 hpf (Fig. 2*D*), suggesting a segregating model of OPC and motor neurons, in which glial fate was restricted prior to cessation of motor neuron generation (24).

For the motor neuron lineage, we found a dynamic transcriptome change along the trajectory. *dlb*, *insm1b*, and *neurod4* were highly enriched in this cluster pri-MN but gradually decreased as cell fate was determined (from motor neuron_1 to motor neuron_2). *Isl2b* and *islr2* were enriched in motor neuron_1 but were decreased in motor neuron_2. *Tac1* and *chga*, however, were significantly upregulated in motor neuron_2 (Fig. 2*J*). *In situ* hybridization confirmed the expression of *insm1a* as well as this novel motor neuron marker *Tac1* (Fig. 2, *L*–*N*). Consistently, the enriched pathways of highly expressed genes switched from neuronal specification to axongenesis and synaptogenesis from pri-MN to motor neuron_1 and motor neuron_2, respectively (Fig. 2*K*; GO term enrichment and their related genes in each cell cluster were available in Supplemental Data 2). Therefore, sc-RNAseq revealed that both oligodendrocytes and motor neurons underwent a complex molecular developmental transition from intermediate precursors into mature cells.

### Identification of *olig2*^*+*^ radial glia and interneuron progenitors

**Figure 3:**
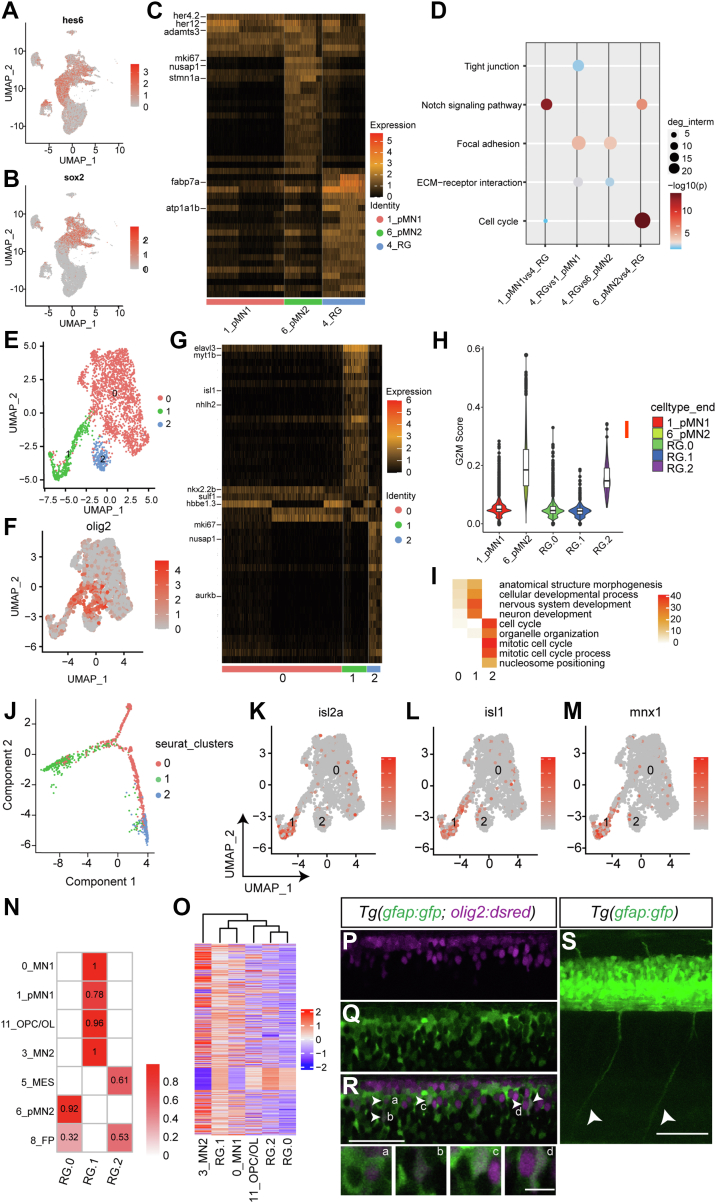
**Identification of neural progenitors for a second motor neuron lineage.***A* and *B*, the expression of neural progenitor markers *hes6* and *sox2* by UMAP. *C*, enriched markers in the radial glia compared to the pMN progenitors. *D*, enriched pathways of differential genes in radial glia and pMN progenitors. *E*, subclustering of the radial glia by UMAP. *F*, the expression of *olig2* in radial glia subpopulations by UMAP. *G*, enriched markers of radial glia subpopulations. *H*, proliferation scores (G2M scores) of subpopulations in the radial glia. *I*, major enriched pathways in radial glia subpopulations. *J*, lineage inference analysis of subpopulations in the radial glia. *K*–*M*, the expression of motor neuronal markers in subpopulations of radial glia. *N*, singleR analysis between radial glia subpopulations and other representative cells. *O*, heatmaps showing gene expression in motor neurons and in subpopulations of radial glia. Selected genes were expressed in at least 60% of MN1 or MN2. *P*–*R*, *olig2+* cells are sometimes colabeled with GFAP. Confocal images of whole-mount *Tg(olig2:dsRed; gfap:GFP)* embryos at 72 hpf, lateral view. Images from a single confocal slice. Notice that dsRed^+^ nuclei are surrounded by the GFP driven by the *gfap* promoter (*arrows*). *Boxes* a–d showing the enlarged images in the *R*. *S*, motor neuron projections were observed in *Tg(gfap:GFP)*. *Arrows* denote the projections from the spinal cord to the periphery. Dorsal top, caudal left. The scale bar represents 50 μm in (*R*) and (*S*). The scale bar represents 10 μm in d. hpf, hour post fertilization; UMAP, Uniform Manifold Approximation and Projection.

In mammals, radial glia can function as neuroepithelial progenitors in the central nervous system and are the primary sources for neurons and glia (25). In zebrafish, though radial glia have been identified as a major source for neurogenesis after injury in the larval and adult zebrafish (26, 27, 28), the characteristics of radial glia in early development are not well understood. Similar to mammals, radial glia in zebrafish were also enriched in neural progenitor markers, for example, *sox2* and *hes6*, indicating their identity as progenitors in early zebrafish development (Fig. 3, *A* and *B*). In contrast to pMN progenitors, radial glia were highly enriched with *gfap*, *her4*.*1*, and *fabp7*, as well as *atp1b1a* and *atp1a1b* (Supplemental Data 1 and Fig. 3*C*). The genes that were differentially expressed in radial glia *versus* those in pMN_1 or pMN_2 clusters were enriched in focal adhesion and ECM–receptor interactions, providing evidence for marked morphological and functional differences in radial glia (Fig. 3*D*).

Radial glia were further classified into three subclusters (Fig. 3, *E* and *F*). Marker genes retrieved in each subcluster revealed that subpopulation 1 was highly enriched with *elavl3* and *nhlh2*, while subpopulation 2, with the highest G2M proliferation score (G2M pathway scores based on G2/M phase markers), was enriched for *mki67*, *nusap1*, and *aurkb* and enriched in the pathways of mitotic cell cycles (Fig. 3, *G*–*I* and Supplemental Data 4). The transcriptional profiles and pseudotemporal ordering of subpopulation 0 were in between those of subpopulation 1 and 2 (Fig. 3, *G* and *J*). These markers indicate that subpopulation 1 is comprised of neurons, while subpopulation 2 is composed of proliferating radial glia, another source of *olig2*^+^ progenitors. The transgenic line *Tg(gfap:EGFP)* was used to label the nuclei and extended process of radial glia (15). In this transgenic line *Tg(gfap:EGFP; olig2:dsRed)*, we observed a few *dsRed*^+^ cells surrounded by GFP (Fig. 3, *P*–*R*), evidence that *olig2* is enriched in subpopulations of radial glia (Fig. 3*F*). Monocle analysis suggests a developmental trajectory from proliferating radial glia to neurons, with subpopulation 2 at the start and subpopulation 1 at the end.

Interestingly, subpopulation 1 in the radial glia was enriched for multiple motor neuronal markers, for example, *isl1*, *isl2a*, and *mnx1* (Fig. 3, *K*–*M* and Supplemental Data 4). The SingleR transcriptome profiling analyses (29) revealed significant similarities between subpopulation 1, MN_1, and MN_2, confirming the motor neuronal features of subpopulation 1 (Fig. 3*N*). Gene expression patterns between subpopulation 1 and motor neurons were highly comparable as shown by heatmaps (Fig. 3*O*). In the transgenic line *Tg(gfap:EGFP)*, motor neuronal projections from the spinal cord to the periphery were observed (Fig. 3*S*). Therefore, single cell analysis suggests a distinct lineage of neurons enriched for motor neuronal markers, derived from *olig*2+ progenitors (radial glia), which is distinct from the previously known OPC/motor neuron lineages.

**Figure 4:**
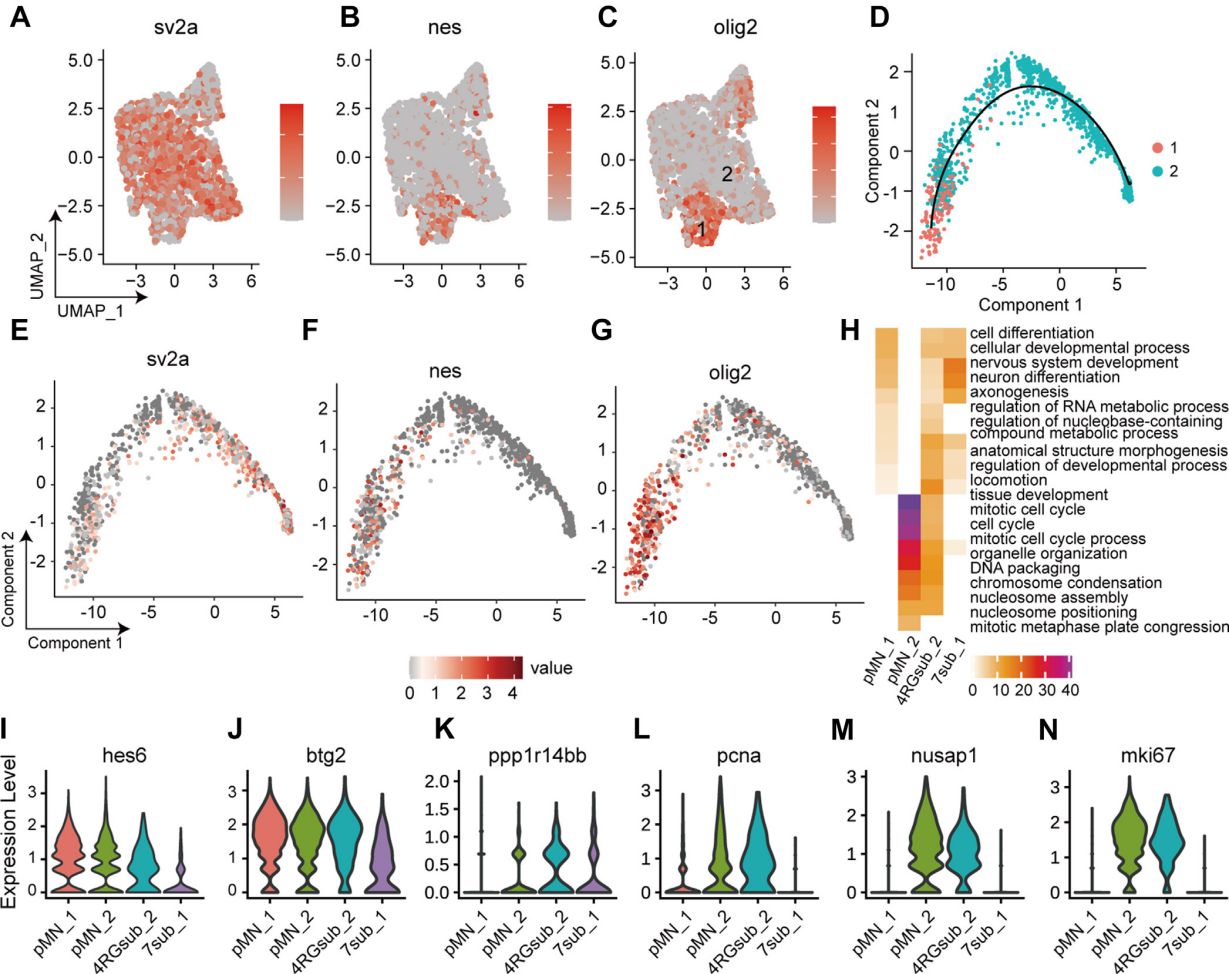
**Identification of neural progenitors for interneurons.***A*–*C*, the expression markers of *sv2*, *nestin* (progenitor markers), and *olig2* in Interneuron_7 by UMAP. *D*–*G*, trajectory lineage inference of subpopulations (1 and 2) in interneuron_7 reveals the developmental trajectory from the *olig2*^+^/*nestin*^+^ cells to *sv2*^+^ neurons. *H*, enriched Go terms for four distinct neural progenitors. *I*–*N*, violin plots showing the expression neural progenitor or proliferation markers. UMAP, Uniform Manifold Approximation and Projection.

In zebrafish, interneurons have been observed among the progeny of pMN progenitors (7). However, the lineage relationship between motor neurons, OPCs, and interneurons was unclear. Interestingly, we observed that a rare subpopulation in interneuron_7 was enriched with *nes* and *olig2* instead of *sv2* (Fig. 4, *A*–*C*), indicating that it is a distinct progenitor population. Lineage trajectory analysis reveals that this subpopulation can develop into mature neurons, a distinguishing feature of which is the expression of *sv2* (Fig. 4, *D*–*G*).

When we analyzed all these progenitors together, we found they share common progenitor genes, including *ppp1414bb*, *hes6*, and *btg2*, confirming the progenitor properties of subpopulation 1 in interneuron_7 (Fig. 4, *I*–*K*). However, unlike the pMN_2 and radial glia, subpopulation 1 in interneuron_7 and pMN_1 are not enriched for cycling markers, including *pcna*, *nusap1*, and *mki67*, consistent with their function in neuronal differentiation (Fig. 4, *H* and *L*–*N*). In summary, our single cell analysis revealed the heterogeneity of *olig2*^+^ progenitors and their distinct lineages (Fig. S4).

### Molecular programming for the MN and OPC lineages

**Figure 5:**
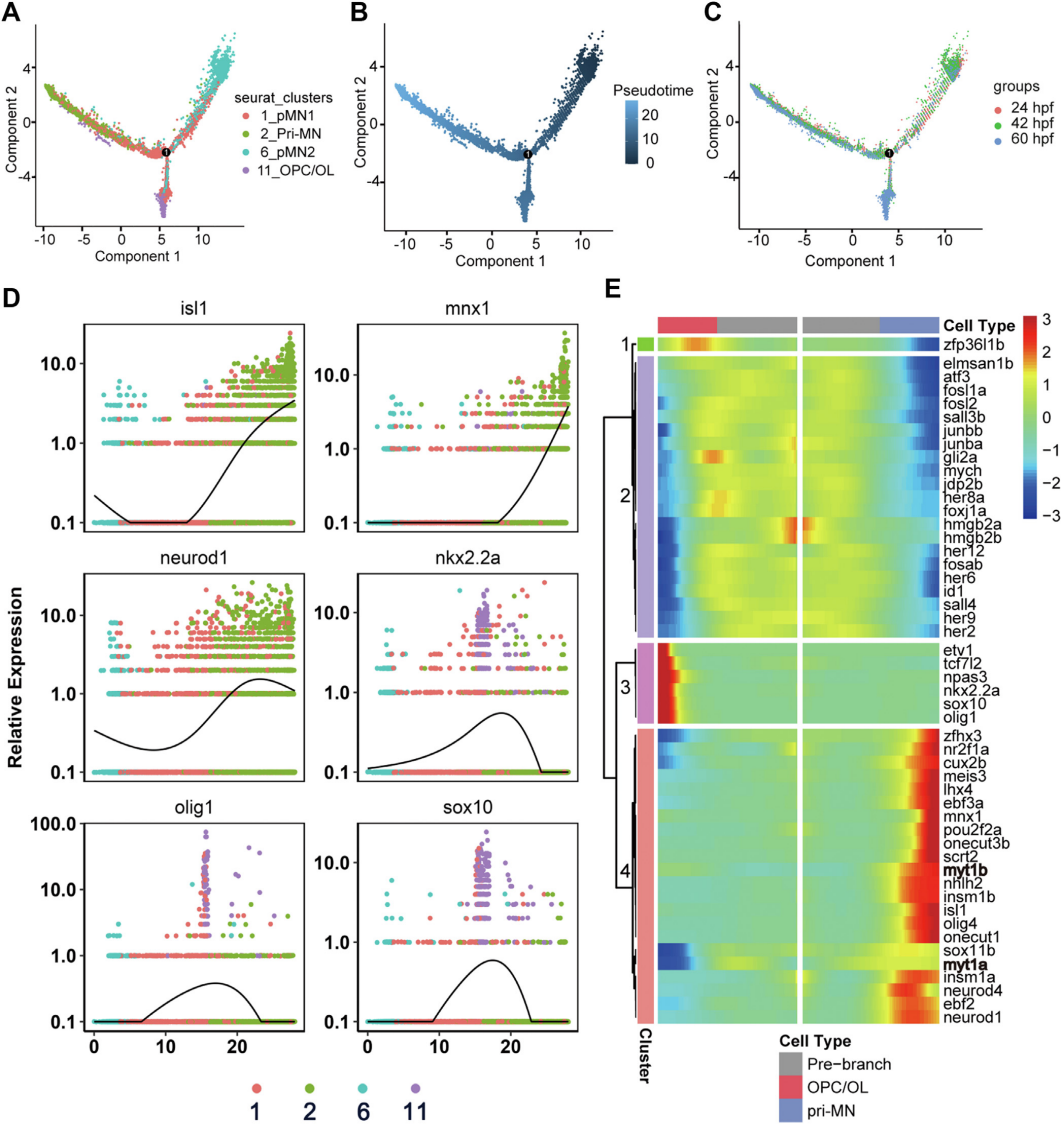
**Lineage and transcription factor analysis for motor neuron and OPC lineages.***A*, monocle showing the putative developmental processes of progenitors or precursors in the pMN domain. Each *dot* represents an individual cell. Bifurcation of pMN progenitors into motor neuron (2) and OPC (11) lineages is shown. *B*, cells in (*A*) are shown in pseudotime. *C*, the temporal distribution of progenitors or precursors in the pMN domain. *D*, expression of known markers along the motor neuron or OPC lineage ordered in pseudotime. Note that *isl1*, *mnx1*, and *neurod1* are enriched in the motor neuron branch, but *nkx2.2a*, *olig1*, *sox10* are enriched in the OPC branch. *E*, heatmaps of the most differentially expressed transcription factors prebranching, in the motor neuron and OPC lineages. The color *red* denotes upregulation; *blue* denotes downregulation of genes. OPC, oligodendrocyte progenitor cell.

pMN progenitors are known as sources for motor neurons and OPCs. In line with this, the trajectory inference analysis software Monocle (30) revealed that the pri-MN and OPC lineages branched from the pMN_1 and pMN_2 clusters (Fig. 5, *A*–*C*). Therefore, understanding the molecular programs that underlie the transition from pMN progenitors to the OPC or pri-MN will elucidate more precise cell fate decisions are important for lineage determination. Temporal changes in gene expression for MN and OPC fate determination were identified (top 500 genes were available in Supplemental Data 5). We selected transcription factors among the top genes that biased the fates of pMN progenitors for further analysis (Fig. 5, *D* and *E*), based on the critical roles of transcription factors in cell fate determination.

For the transcription factors, we observed four distinct gene expression patterns along the MN and OPC trajectories: (1) a pattern of a decrease in the MN but not in the OPC trajectory (Cluster 1); (2) a pattern of a decrease in either the MN or the OPC trajectory (Cluster 2); (3) a pattern of an increase in the OPC trajectory (Cluster 3); (4) a pattern of an increase in the MN trajectory (Cluster 4). Cluster 2 included *hmgb2a*, *her6*, and *her2*, which are known for counteracting cell differentiation programming and maintaining the stemness of neural stem cells or progenitors. Interestingly, genes previously identified as “regeneration-associated genes”, such as *junba*, *atf3*, and *junbb*, were also included in this cluster. This indicates that a complex dedifferentiation and neuron reprogramming process occurs in regeneration. In Cluster 3, transcription factors such as *olig1*, *sox10*, and *nkx2.2a* were included, consistent with their role in OPC lineage determination (Fig. 5, *D* and *E*). In Cluster 4, multiple transcription factors known for motor neuron specification, including *mnx1*, *lhx4*, *isl1*, *insml1a*, *insm1b*, and *neurod1*, were identified and defined as “MN transcription factors” (Fig. 5, *D* and *E*). These results were largely consistent with the differentially expressed gene (DEG) analysis between pri-MNs and OPCs, which revealed multiple differentially expressed genes or transcription factors for motor neuron or OPC fate determination (Fig. S5, *A* and *B*), indicating the reliability of the different algorithms.

### The role of *myt1* in the neuronal lineage

**Figure 6:**
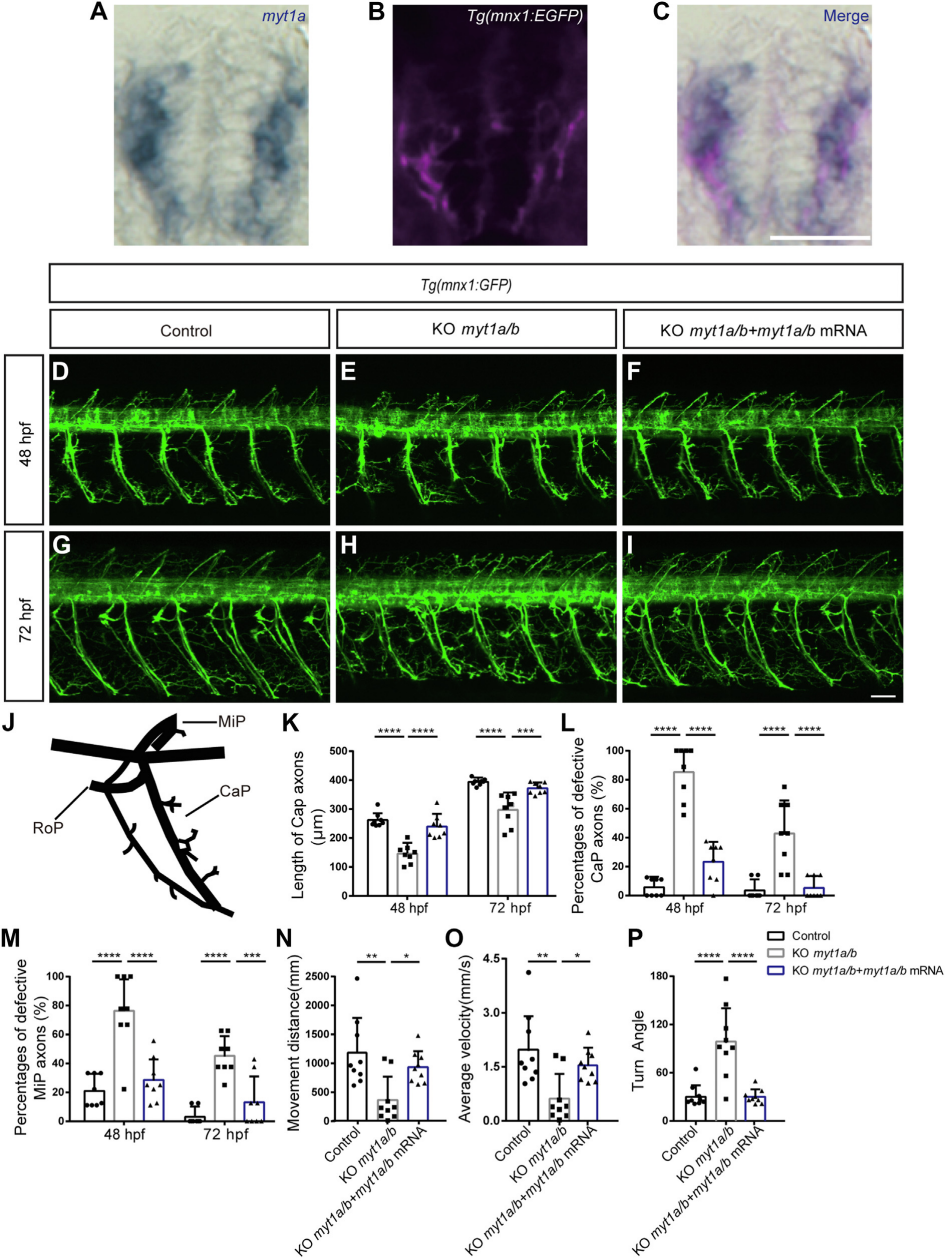
***Myt1* is necessary for the development of motor neurons.***A*–*C*, transverse sections of spinal cord showing *myt1a* RNA (*blue*) in *Tg(mnx1:EGFP)* (*pink*) embryos. Dorsal top, rostral left. The scale bar represents 25 μm. *D*–*I*, confocal imaging analysis of primary motor neurons in control, KO *myt1a/b*, and mRNA rescue groups at 48 hpf (*D*–*F*) and 72 hpf (*G*–*I*) *Tg(mnx1:GFP)*. *Arrowhead* denotes round-shaped and undifferentiated neuronal cells. The scale bar represents 50 μm. *Arrows* denote missing or shortened MiP or CaP axons. *Dashline* in (*H*) shows CaP axons at 72 hpf. Enlarged images of (*D*–*I*) are available at Fig. S7. *J*, schematic diagram of three primary motor axon projections (CaP, MiP, and RoP) in zebrafish. *K*, the length of CaP axons of zebrafish in the control group, in the KO *myt1a/b* group, and in the mRNA rescue group at 48 hpf (n = 8 in each group, ∗∗∗∗*p* < 0.0001, one-way ANOVA followed by Turkey’s test) and 72 hpf (n = 8 in each group, ∗∗∗∗*p* < 0.0001, ∗∗∗*p* < 0.001, one-way ANOVA followed by Turkey’s test). *L*, the percentages of defective CaP axons in the control group, the KO *myt1a/b* group, and in the mRNA rescue group at 48 hpf (n = 8 in each group, ∗∗∗∗*p* < 0.0001, one-way ANOVA followed by Turkey’s test) and 72 hpf (n = 8 in each group, ∗∗∗∗*p* < 0.0001, one-way ANOVA followed by Turkey’s test). *M*, the percentages of defective MiP axons in the control group, the KO *myt1a/b* group, and mRNA rescue group at 48 hpf (n = 8 in each group, ∗∗∗∗*p* < 0.0001, ∗∗∗*p* < 0.001, one-way ANOVA followed by Turkey’s test) and 72 hpf (n = 8 in each group, ∗∗∗∗*p* < 0.0001, ∗∗∗*p* < 0.001, one-way ANOVA followed by Turkey’s test). *N* and *O*, distance and velocity analysis in the control group, the KO *myt1a/b* group, and mRNA rescue group in 5 dpf larvae (n = 9, respectively, ∗∗*p* < 0.01, ∗*p* < 0.05, one-way ANOVA followed by Turkey’s test) by the video tracking software EthoVision XT. *P*, turn angle in the control group, the KO *myt1a/b* group, and mRNA rescue group in 5 dpf larvae (n = 9, ∗∗∗∗*p* < 0.0001, one-way ANOVA followed by Turkey’s test). All data were shown as the mean ± SD. ∗, ∗∗, ∗∗∗, and ∗∗∗∗ denote *p* < 0.05, *p* < 0.01, *p* < 0.001, *p* < 0.0001, respectively. n.s., not significant. hpf, hour post fertilization.

We noticed that the transcription factors *myt1a* and *myt1b* (two isoforms of *myt1* in zebrafish) stood out in Cluster 4 (Fig. 5*E*). *Myt1b* was significantly increased in the MN trajectory, while *myt1a* was increased in the MN trajectory but decreased in the OPC lineage. In line with this, we found that both *myt1a* and *myt1b* were enriched in motor neurons of zebrafish (Figs. 6, *A*–*C* and S6, *A*–*C*). Myt1, myelin transcription factor 1, was identified as a critical molecule in oligodendrocyte differentiation (31), but its role in spinal cord circuit formation has not been well characterized.

To determine the role of *myt1* in motor neuron specification, we used CRISPR/Cas9-mediated gene disruption of *myt1a* and *myt1b* (Fig. S6, *D* and *E*). We coinjected *myt1a* and *myt1b* sgRNAs and Cas9 protein into one-cell stage embryos and found up to 100% mutagenesis in either *myt1a* or *myt1b* DNA amplicons of F0 embryos (n = 11 and 13, respectively), as shown by Sanger sequencing at 48 hpf (Fig. S6, *F* and *G*). To test the extent of mutagenesis, we randomly selected three F0 samples and cloned 11 and 13 individual PCR amplicons in *myt1a* and *myt1b*, respectively. All clones had mutations (deletions, insertions, or nucleotide substitutions), and many of them led to an out-of-frame protein product (Fig. S6, *H*–*K*). Testing the stability of CRISPR, we found that the mutagenesis perdured for at least 3 months (Fig. S6*M*). In addition, the disruption of the myt1 gene was confirmed by quantitative RT-PCR, which showed a significant reduction in either *myt1a* or *myt1b* mRNAs compared to the WT levels (Fig. S6*L*).

To analyze the development of motor neurons, we used the transgenic line *Tg(mnx1:EGFP)* in which EGFP was driven by the motor-neuron–specific transcription factor *mnx1*. At 48 hpf, when many motor neurons have been actively generated, compared to controls, most motor neurons in *myt1* mutant had round cell bodies and lacked processes, which was considered to be an undifferentiated state (32) (Figs. 6, *D* and *E* and S7, *A* and *B*). In zebrafish, three primary motor neurons exist in the spinal cord: caudal primary (CaP) motor neurons, middle primary (MiP) motor neurons, and rostral primary (RoP) motor neurons, which innervate the ventral trunk musculature, dorsal trunk musculature, and muscle fibers in between, respectively (Fig. 6*J*). We viewed that many CaP or MiP axons were missing or shortened (Figs. 6, *E* and *K*–*M* and S7*B*). The average length of the CaP axons was significantly shortened (Fig. 6, *E* and *K*), and the defective CaP axons were dramatically increased in *myt1* mutant zebrafish (Fig. 6*L*). Approximately, 80% of MiP axons were missing or shortened in mutants (Fig. 6*M*). Most RoP axons in the mutant were observable. However, it was difficult to determine their start and end points, which precluded further quantitative analysis.

To exclude the possibility of developmental delay induced by CRISPR injection, we verified motor neuron specification at 72 hpf (Fig. 6, *G*–*I*), a time point when motor neuron generation is complete. In *myt1* mutants, the average length of CaP axons was significantly shortened (Figs. 6, *G*, *H* and *K* and S7, *D* and *E*), and ∼40% of CaP axons had defects in their anterior lateral projections (Fig. 6*L*). Approximately, 50% of MiP axons were missing or shortened (Fig. 6*M*). The motor neuron developmental defects induced by *myt1* CRISPR were rescued by coinjection of *myt1* mRNA (Figs. 6, *F*, *I* and *K*–*M* and S7, *C* and *F*). When we tested the role of *myt1* in OPC development, we did not find any obvious OPC specification defects (Fig. S8, *A*–*F*). This indicates that the maturation of motor neurons were not obligatory for the generation of OPCs.

We also tested the behavioral outcomes of *myt1* mutagenesis in 5 days larvae. *Myt1* mutants have disrupted locomotor activity, as shown by a reduction in distance and velocity (Fig. 6, *N* and *O*). Interestingly, we also found an increase in the turn angle, which was defined as the orientation of the head relative to the body when turning (Fig. 6*P*). These indicate that both locomotion and motor coordination were affected in *myt1* mutants. Interestingly, these defects can be rescued by coinjection of *myt1* mRNA (Fig. 6, *N*–*P*).

### The role of *lbr* in neural progenitor maintenance

**Figure 7:**
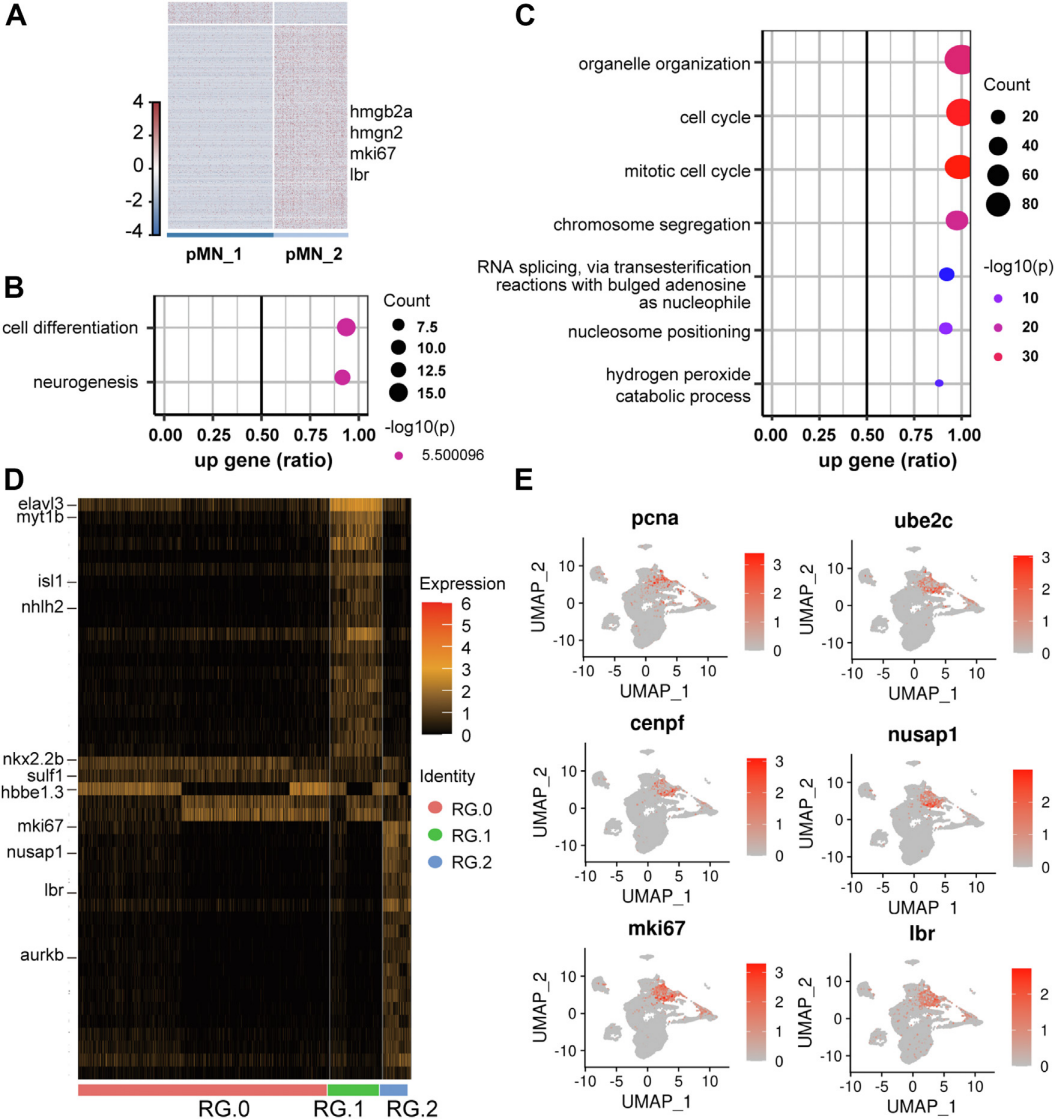
***lbr* is enriched in proliferative neural progenitor or radial glial cells.***A*, DEG analysis between pMN_1 and pMN_2. *B* and *C*, upregulated (*B*) and downregulated (*C*) pathways in pMN_1. *D*, enriched genes in subpopulations of radial glia. Notice that *lbr* is enriched in proliferative radial glia (RG_2). *E*, UMAP visualization of cell cycle–related genes. Panel *E*'s clusters are identical to those in Figure 1*B*'s. Notice that the expression of *lbr* is highly correlated with other cell cycle–related genes. DEG, differentially expressed gene; UMAP, Uniform Manifold Approximation and Projection.

The pMN_1 and pMN_2 clusters were enriched for *olig2* and developed into two lineages, indicating these were pMN progenitor cells. From lineage analysis, both motor neuron and OPC restricted precursors appeared to be derived from the cluster pMN_1 rather than the pMN_2 cluster (Fig. 2*A*). By DEG analysis, we found that multiple known proliferative markers were downregulated in pMN_1 compared to pMN_2, including *ki67*, *hmgb2a*, and *hmgb2b* (Fig. 7*A*). Notably, the transition from pMN_2 to pMN_1 was accompanied by dramatic downregulation of expression of most genes and upregulation in fewer genes (Fig. 7*A*). GO analysis shows that genes downregulated in pMN_1 were enriched in cell cycle and chromosome-remodeling–related pathways (*e.g.*, chromosome segregation), indicating a decreased proliferative capacity in pMN_1. GO analysis for upregulated pathways in pMN_1 *versus* pMN_2 revealed that cell differentiation and neurogenesis pathways were enriched (Fig. 7, *B* and *C*).

Taken together, all of these results show that the pMN_2 cluster represents proliferating states while the pMN_1 cluster corresponds with differentiating states of neural progenitors; the transition between these two states is accompanied by dynamic transcriptional profiling changes, especially with the downregulation of genes involved in the cell cycle or proliferation. Our results indicate that proliferation of progenitors counteracts differentiation; when the cell proliferation capacity is reduced, differentiation occurs. The number of downregulated DEGs in the differentiation state, which were enriched in chromatin remodeling and proliferation, consistently far exceeded the number of upregulated DEGs in our progenitor DEG analysis (Fig. 7, *A*–*C*).

By DEG analysis, we found that multiple known proliferative markers were downregulated in pMN_1 compared to pMN_2, including *ki67*, *hmgb2a*, and *hmgb2b* (Fig. 7*A*). Several novel feature genes, for example, the *lbr* (lamin B receptor), were also dramatically decreased (Fig. 7*A*). Interestingly, *lbr* was also enriched in the proliferative radial glia (RG_2, Fig. 7*D*). The correlated expression between *lbr* and other proliferation markers such as *pcna* (Fig. 7*E*) indicates that *lbr* might be important in the fate transition of neural progenitors.

LBR is known for its role in neutrophil differentiation; a deficiency in LBR led to improper neutrophil nuclear lobulation, which is a feature of the Pelger-Huët anomaly (33). However, the role of the LBR in neural progenitor cells has not been explored. Loss of the LBR in zebrafish led to severe developmental defects (34), which precluded further analysis.

**Figure 8:**
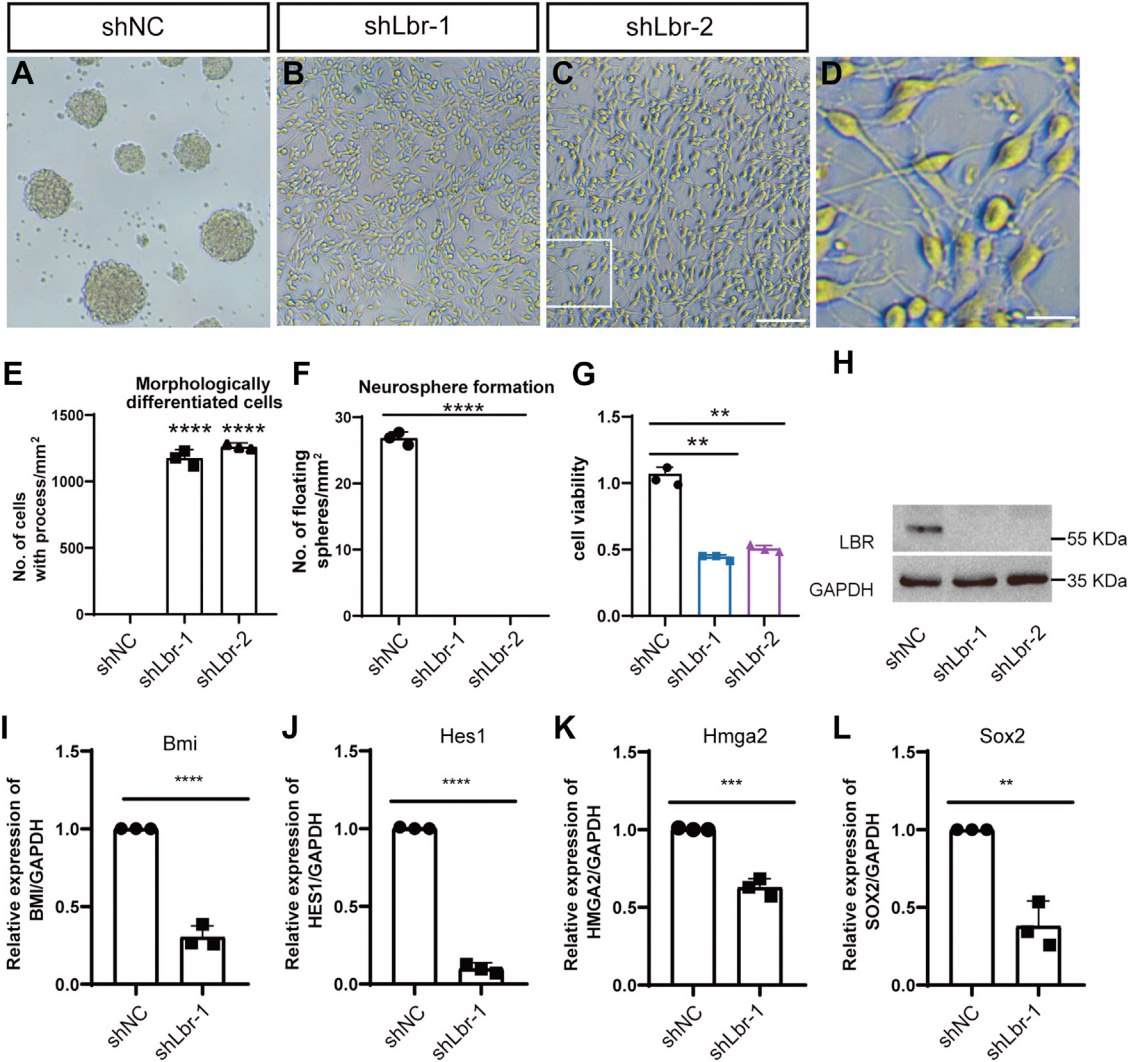
**Cell culture of neural stem cells dissected from mouse brain.***A*–*C*, morphology of cells transfected with a scrambled control (shNC, *A*) and Lbr knock down lentivirus (shLbr-1 and shLbr-2, *B* and *C*). The scale bar represents 100 μm. *D*, the enlarged images from the boxed area in (*C*). The scale bar represents 25 μm. *E* and *F*, quantification of cells with processes (*E*) and the number of neurosphere formation (*F*). *G*, CCK8 analysis of cells transfected with Lbr lentivirus. *H*, Western blot reveals a dramatic decrease of LBR expression in cells transfected with shLbr-1 or shLbr-2. *I*–*L*, qPCR analysis of stemness-related genes. Data were shown as the mean ± SD. ∗∗, ∗∗∗, and ∗∗∗∗ denote *p* < 0.01, *p* < 0.001, *p* < 0.0001, respectively.

To test the role of the LBR in neural stem/progenitor cells, we isolated them from fetal rat spinal cords and transduced them with either a scramble control (shNC) or two individual lentivirus LBR shRNAs targeting two different coding regions of the Lbr (shLbr-1, shLbr-2). Both shLbr-1 and shLbr-2 were robust in reducing LBR expression, with an efficiency up to 100%, as shown by Western blot (Fig. 8*H*).

**Figure 9:**
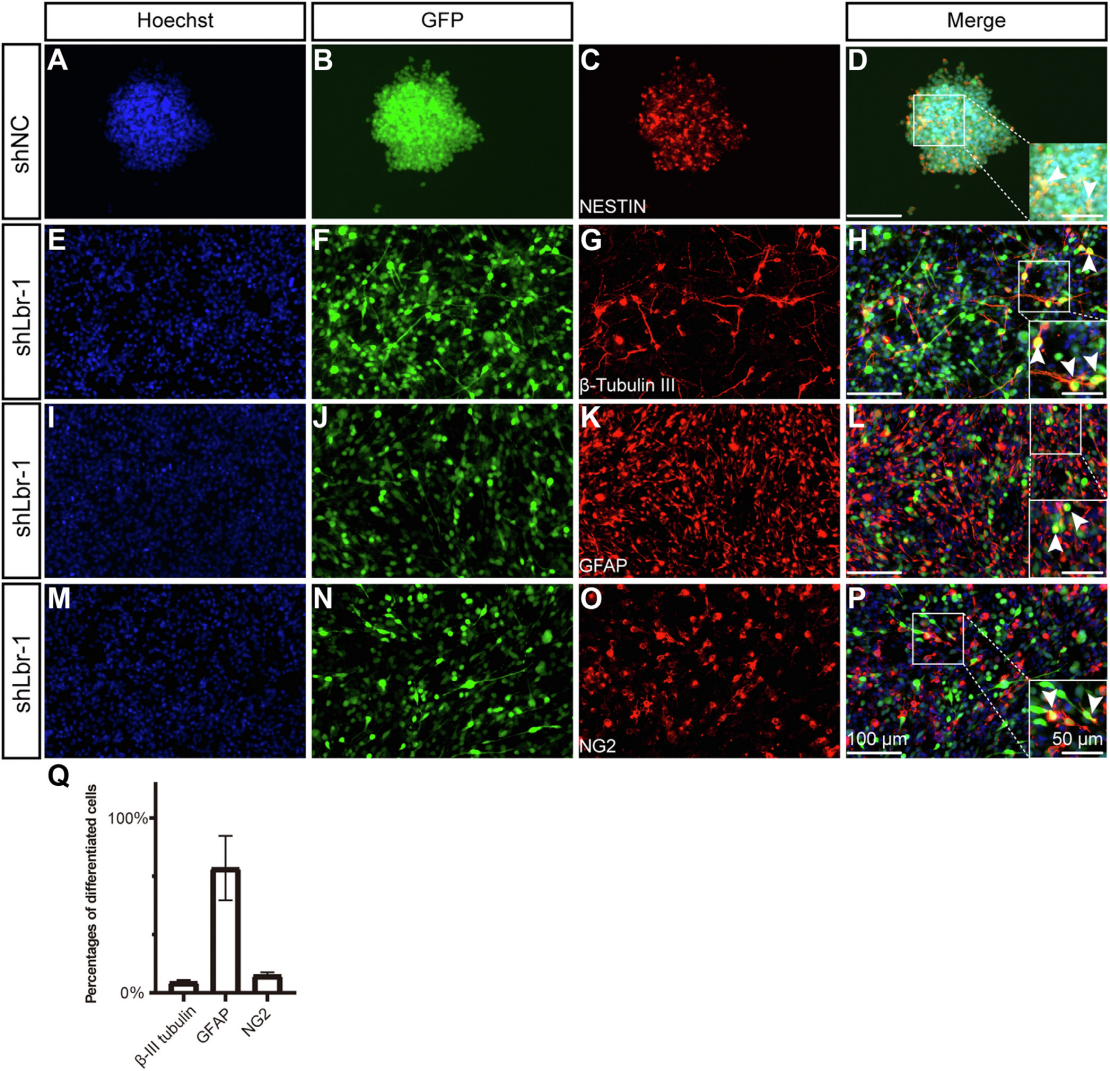
**Characterization of cells transfected with Lbr lentivirus.***A*–*D*, neurospheres formed in shNC. *E*–*P*, cell morphology and antibody staining in cells transfected with Lbr lentivirus. Hoechst (counterstaining for cell nuclei), GFAP (an astrocytic marker), NG2 (an OPC marker), and β-III tubulin (a neuronal marker) are used. The scale bar represents 100 μm in (*P*). The scale bar represents 50 μm in enlarged images. GFP is carried by the lentivirus. *Arrowheads* represent representative colabeling between GFP and β-III tubulin, NG2, or GFAP. Not all colabeled cells are marked. *Q*, quantification of differentiated cells transfected by Lbr lentivirus. Data were shown as the mean ± SD. OPC, oligodendrocyte progenitor cell.

Two days after seeding single cells, neurospheres formed readily in the group transduced with the control shRNA (Fig. 8*A*), and their identity was confirmed by immunofluorescent staining with nestin (Fig. 9*C*), a neural stem/progenitor cell marker. Conversely, few neurospheres were detected in neural stem cells transduced with either shLbr-1 and shLbr-2. Instead, these cells adhered to the plates, with significant changes in morphology (Fig. 8, *B*–*D*). Greater than 98% of these adherent cells bore processes, strongly resembling differentiated cells (Fig. 8, *B*–*D*). Consistently, multiple stemness-related genes including Sox2, Hmgn2, Hes1, and Bmi were significantly downregulated when transfected with shLbr (Fig. 8, *I*–*L*). In addition, cell number was reduced when the LBR was knocked down as shown by CCK8 analysis (Fig. 8*G*).

To characterize the identity of these cells transfected with shLbr, we used several markers: GFAP for astrocytes, β-III tubulin for neurons, and NG2 for OPCs. Cells that lost the LBR stained positive for either GFAP, β-III tubulin, or NG2 (Fig. 9, *A*–*Q*). Therefore, the loss of the LBR was sufficient for spontaneous differentiation even in the neural stem medium. To summarize, loss of the LBR led to spontaneous differentiation of the stem cell population accompanied by a weakened self-renewing capacity. This observation highlights the capability of scRNA to not only decipher cell states or properties but also to offer a powerful approach for detecting transcriptional regulators in cell fate determination.

### Web-based tools for exploration

Cell cluster definition is not well established in zebrafish due to the relative paucity of databases, and zebrafish have unique cellular expression signatures compared to mammals. We generated a web-based tool for our scRNA-seq datasets, including not only *olig2*^+^ cells (progenitors, precursors, as well as multiple states of motor neurons) but also interneurons and other cells sorted by FACS. With this interface, users can visualize gene transcription levels and patterns and/or explore highly expressed genes in each individual cluster. This tool is available at: https://nantongneurokeylab.shinyapps.io/cell_browser/.

## Discussion

Our study, utilizing scRNA-seq, examines *olig2*^+^ multiple progenitors in the spinal cord, which differ in fates and transcriptome signatures. We also characterize lineage-enriched factors involved in regulating zebrafish pMN progenitor development and experimentally demonstrate that *myt1* is necessary for motor neuron development and that *lbr* is important for the maintenance of stemness in neural stem/progenitor cells. Our study suggests a new interpretation for the apparent conflicts in previous research regarding the motor neuron and OPC lineages and provides molecular programming for neural progenitor fate transitions.

We find that the *olig2*^+^ cells are more heterogeneous than expected, because they include not only known pMN progenitors for motor neurons and OPCs but also progenitors for interneurons and *olig2*^+^ radial glia (dividing cells). *olig2* is enriched in one subpopulation of interneurons (Interneuron_7), whose lineage seems disconnected from the pMN progenitors (Fig. 2*A*). Though the pMN domain is defined by the expression of *olig2*, cells profiled by virtue of expressing an *olig2*^+^ reporter may not only derive from the pMN domain. The Olig2-Cre and Olig2-reporter can drive expression in interneurons (35), which could be due to transient expression of Olig2 in interneurons prior to cross-repressive interactions sharpening the boundary of the pMN and adjacent interneuron domain. Interestingly, radial glia, as neural progenitors, may also generate neurons enriched for motor neuronal markers. The motor neuronal projections labeled with the *gfap* reporter show up after 48 hpf and have few branches, indicating that these are secondary motor neurons. This is consistent with the finding that *gfap*^+^ cells can colabel with gata2^+^ secondary motor neurons (36). This may explain why previous results had differing conclusions as to the identity of cell lineages traced by this single *olig2* reporter (3, 7). Our study shows that with only a single marker/reporter, *olig2*, for example, it is hard to distinguish different subpopulations of progenitors, and this may lead to inaccurate lineage tracing. Understanding the heterogeneity of olig2+ cells is crucial for obtaining the desired cell lineage. Our interactive website offering signature genes in each subpopulation will give researchers access to a powerful tool to develop more specific markers for cell labeling. In future studies, it may be necessary to integrate multiple techniques including newly developed DNA-barcoding technologies to decipher the lineages or fates of neural progenitors (37).

The characteristics of pMN progenitors identified provide an in-depth understanding of previous findings regarding the lineage analysis of glia and neurons. Both common and distinct progenitor models have been proposed for the motor neurons and oligodendrocytes. Analyses of the progeny of clones isolated from neural tissue in mice, chick, and zebrafish support a common progenitor model, in which progeny clones contain lineage cells for both motor neurons and OPCs (6, 7, 38). However, live imaging analysis suggests a distinct progenitor model (3). Interestingly, to some extent, our single cell data regarding the diversity of *olig2*^+^ progenitors with two different motor neuron lineages can fit into both of these two apparently conflicting models. In addition, the existence of progenitors for radial glia, interneurons, OPCs, and motor neurons is consistent with the finding that multiple cell types can arise from the same clone of *olig2*^+^ cells (6, 7, 38). Similar to recent work (24), we also find pri-OPCs appearing as early as 28 hpf, when motor neurons are actively generated. This seems to support a “segregating” model in which motor neurons and oligodendrocyte precursors segregate early (39). However, the ratio of this subpopulation was much lower than that of the pri-MN at early stages of development, which may explain why a single *olig2*+ clone cell mainly generates neurons instead of OPCs (3, 7). The percentages of progenitors or precursors are temporally dynamic, with pMN progenitors decreasing with time, suggesting a more restricted fate at later stage. Therefore, the timing of analysis of progenitors and their progeny is also an important factor to consider. Our study also reveals that pri-MNs have a low proliferation score, which is consistent with the finding that progenitors generating motor neurons seldom divide (3).

Though the diversity of pMN progenitors has been recently characterized (24), the transcriptome features and molecular programming for lineage priming are not clear. In concert with fate transition, we find that progenitor markers and genes enriched in metabolic pathways vary. Though these might be consequences of fate determination, it would be interesting to explore whether different molecules/regulators are deployed to maintain undifferentiated states in the progenitors or precursors and whether metabolic cues can interact with transcription factors driving fate determination, since they can also act as key regulators for fate determination (40). Lineage analysis of the single-cell transcriptome deepens our understanding of molecular programs driving neuron or OPC fate determination and elucidates lineage-enriched factors. Early studies focused on the role of myt1 in oligodendrocyte differentiation (31), and its role in neuron specification has been recognized recently (41). Our work shows that myt1 is upregulated in motor neuron precursors, indicating that it may function in neuron fate determination. Though Myt1 was found important for oligodendrocyte differentiation in mice (31), we did not find that myt1 deficiency led to OPC specification defects. For future work, it would be interesting to dissect out the specific role of myt1 in OL lineage development (including OPC specification and oligodendrocyte maturation) in both mice and zebrafish. It may be also interesting to explore how this transcription factor works with general neuronal transcription factors, for example, neurogenin 1 and neurogenin 2, and motor neuron-specific transcription factor, for example, insm1 or insm2. In addition, we noticed that myt1 is highly enriched in neuronal cells, including the motor neuron lineage and interneurons (Supplemental Data 1), indicating its critical role in neuronal fate determination. One limitation of our study is that the cell-autonomous roles of myt1 in motor neurons were not validated due to technical constraints. The MYT1 family includes three proteins: MYT1, NZF3, and MYT1l (MYT1-like). MYT1l has been implicated in neuronal reprogramming either alone or in combination with the transcription factors ASCL1 and BCL2 (42, 43). Neuronal reprogramming has been applied to generate neurons in many diseases and pathological states, including spinal cord regeneration, Parkinson’s Disease, and Alzheimer’s Disease (44, 45, 46). Therefore, it might be interesting to explore the role of MYT1 in neuronal reprogramming, which may offer new insights into neuronal regeneration.

During neutrophil differentiation, LBR expression is increased and critical for myeloid cell growth and functional maturation (33). The LBR has also been shown to inversely regulate muscle cell differentiation (47), suggesting that it could be a general molecule used by various cell systems to transition to a different cell fate. Loss of Lbr weakened the ability of neural stem cells/progenitors to self-renew, resulting in spontaneous differentiation into neurons or glia in our study. For further work, it would be interesting to explore the cell-autonomous role of LBR in cell stemness. Neural stem cell transplantation has been widely used to generate neurons or glial cells to replace lost cells following trauma or degeneration (48), in which cell expansion *in vitro* and tumorigenesis *in vivo* are challenging issues. Low differentiation and high proliferation rates are critical for stem cells/progenitors in tumorigenesis and self-renewal. Based on the role of LBR found in this study, it may be a potential molecular target for cell transplantation modification.

In conclusion, our work provides insights into the characteristics of progenitors and precursors in the pMN domain, which is essential for understanding the basis of nervous system circuit formation. The identification of critical programming in cell fate determination may also increase our understanding of CNS disorders and may be harnessed for neuronal regeneration.

## Experimental procedures

### Ethics statement

All animal experiments were performed in accordance with the NIH Guide for the Care and Use of Laboratory Animals (http://oacu.od.nih.gov/regs/index.htm). All procedures and protocols were approved by the Administrative Committee for Experimental Animals, Jiangsu Province (Approval ID: SYXK (SU)2007–0021).

### Zebrafish husbandry and collection of embryos

WT and transgenic zebrafish *Tg(olig2:dsred)*^*vu19*^ (14), *Tg(sox10*:*GFP)*^*ba2*^ (49), and *Tg(mnx1:GFP)*^*ml2*^ (50) were obtained from the China Zebrafish Resource Center. Zebrafish feeding and embryo manipulations were conducted according to standard protocols in the Nantong Zebrafish Core Facility. The adults were raised at 28 °C, with 14/10 h light-dark cycles. Embryos were raised in petri dishes at 28 °C.

### Single cell isolation and sorting

The transgenic line *Tg (olig2:dsRed)* was outcrossed to WT fish, and embryos were anesthetized at 28, 42, and 60 hpf with 0.04% MS-222 and dechorionated. Heads were removed rostral to the hindbrain-spinal cord boundary using a 24 G needle. The remaining trunks were deyolked in deyolking buffer (55 mM NaCl, 1.8 mM KCl, and 1.25 mM NaHCO3) and minced with fine scissors. Samples were dissociated with 0.25% trypsin at 28 °C for 30 min followed with 1 mg/ml collagenase II for 20 min. 10% fetal bovine serum was added to terminate the enzymatic reaction. Samples were passed through a 70 μm strainer and sorted with a BD FACS-Aria fusion machine as previously reported (51). FACS was performed in the core facility of Shanghai Institute of Nutrition of Heath, Chinese Academy of Sciences. FACS gating scatter plots with gating strategy was provided in the Fig. S9.

### Single cell capture, sequencing, and data processing

The cell suspension (300–600 cells/μl) was loaded onto a 10× Chromium platform according to the manufacturer’s instructions. Captured cells were lysed, and released RNAs were reverse transcribed into complementary DNAs (cDNAs). cDNAs were amplified and libraries were generated with the Single Cell 3′ Library and Gel Bead kit V3. The libraries were sequenced on an Illumina Novaseq 6000 with at least 100,000 reads per cell (CapitalBio Technology) using 150 bp paired-end runs. The Cell Ranger, downloaded from https://support.10xgenomics.com/single-cell-gene-expression/software/downloads/latest, was used to de-multiplex Illumina output and generate feature-barcode matrices.

### Cell clustering and lineage analysis

Genes detectable in more than two cells were defined as “expressed”. Cells with <1000 genes detected were removed. Counts were normalized to 10,000 and transformed into logarithmic scales. A neighborhood graph was embedded using UMAP displaying the top 4000 highly variable genes across cells. Cells were clustered by the Louvain Algorithm. The trajectory interface of all cell clusters was done using the layout 'fa'. These analyses were performed by the Python-based SCANPY package, designated as PAGA (23). Trajectory lineages of pMN progenitors or precursors were also analyzed with Monocle with built-in R (30). For PAGA analysis, clustering of all cells as well as clustering of subpopulations of pMN progenitors and precursors were performed. For Monocle analysis, only subpopulations of pMN progenitors/precursors were analyzed.

### *Myt1* CRISPR/Cas9

One-cell stage zebrafish embryos from *Tg(mnx1:GFP)*^*ml2*^ (50) outcrossed to WT were injected with 450 pg Cas9 protein (Novoprotein, E365), 225 pg *myt1a*, and 225 pg *myt1b* sgRNAs. SgRNA synthesis was performed as previously reported (52). Briefly, double-stranded DNA for the gRNA was PCR amplified with the template vector pMD 19-T and the following primers were used: *myt1a* sgRNA forward: GGGGGACTCCTGGAACAGGC, *myt1b* sgRNA forward: GGCCGCAGTCTGTCAGGCTG, and universal reverse: AAAAAAAGCACCGACTCGGTGCCAC. Amplicons were purified with the DNA clean & concentrator kit (Zymo Research). Functional sgRNA was transcribed using the MegashortscriptTM kit (Thermo Fisher Scientific) and purified with the Megaclear kit (Thermo Fisher Scientific).

### Verification of mutagenesis generated by CRISPR

Genomic DNA was extracted from 48 or 72 hpf embryos and analyzed as previously described (53). In brief, 50 mM NaOH was used to lyse embryos heated at 95 °C for 30 min. DNA surrounding the *myt1a* and *myt1b* sgRNA regions was amplified with their respective primers: *myt1a* F:GACCACCAGTACTTCAGCGG R:CGGCGATGTCCATGACCTTA; *myt1b* F:AGCATGAAACTGGAGAGCGT R:TCGAAGGCACGGATTGTCTT. The targeted DNA regions were purified and sequenced by Sanger sequencing. To analyze the frequency of mutagenesis, amplified PCR products from F0 embryos were cloned into PCR4.0 TOPO (ThermoFisher). Plasmids from individual colonies were isolated and sequenced by Sanger sequencing (Genewiz, Inc). qRT-PCR was used to detect the mRNA levels of *myt1* in crispants and embryos with Cas9 injected alone. Each reaction was performed in triplicate at 48 hpf. Primers for *myt1a* were forward 5′- TCACAGAGGTCAGAGGTCACT-3′ and reverse 5′-CGTCCTGTCGTCTAAGCTGT-3; and primers for *myt1b* were forward 5′- AACCCCTGGGTGTGATGGAA-3′ and reverse 5′- CTGTTGACATGACCCTGACCA-3.

### Analysis of larval locomotor activity

Larval behavior was tracked at 5 dpf with an Ethovision XT 13.0 equipped with a Basler GenlCam (Basler acA 1300-60) at a rate of 25 images/s. At 5 dpf, larvae were transferred into a 24-well plate with one larva per well. After acclimatization in the DanioVision Observation Chamber (Noldus Information Technology) for 5 min in the dark, swimming behavior was recorded for 10 min. We used a light/dark flash model with 10 s (seconds) light followed by 10 s dark (30 cycles, for a total of 10 min). This model was to evaluate the response of larvae to acute illumination changes. The light/dark program settings of this method were derived from the manufacturer's manual. The parameters we analyzed included total distance moved, average speed, angular velocity, and turn angle. To calculate total distance moved, we inferred distance moved relative to the center. 0.2 mm was set as the threshold value to exclude slight movements that were not considered to be swimming.

### *In situ* probe generation and *in situ* hybridization

*In situ* RNA probes for *olig2*, *mnx1*, *myt1a*, *myt1b*, insm1a, and *tac1* were generated by a plasmid-free approach as reported previously (54). Primers used for probes were listed in Table S1. cDNAs from 2 and 3 dpf embryos were amplified with related primers, and PCR products were purified with the Zymo Research DNA Clean & Concentrator-5 kit. T7 polymerase was used to make digoxigenin-labeled antisense probes for *in situ* hybridization. Whole-mount *in situ* hybridization and fluorescence *in situ* hybridization followed by immunostaining of embryo sections were performed as previously described (53).

### Microscopy and image analysis

Confocal images of transgenic zebrafish were acquired as previously described (52). Live Tg(olig2:dsRed;sox10:EGFP) and Tg(mnx1:EGFP) embryos were mounted lateral side up in low-melt agarose. Confocal z-stacks were taken with the same confocal settings (*e.g.*, the same PMT and imaging speeds). Analysis was performed on max Z projections in ImageJ. Approximately, seven consecutive spinal cord segments in the confocal imaging windows were analyzed, with the most rostral segment over the caudal margin of the yolk sac. To quantify the length of the CaP axons, we imported the images into Imaris Software (Oxford Instruments), traced the axons, and calculated their lengths. In addition, we used ImageJ to calculate the length of RoP and MiP axons. For quantification of the OPC lineage cells, puncta were tabulated with ‘analyze particles’ in ImageJ with a size of 0.01 to infinity and a circularity of 0.01 to 1.00. Experiments were performed blinded to the experimental condition and the observer was blinded to the experimental groups before scoring or measuring.

### Neural stem/progenitor cell isolation and culture

An adult female SD rat, pregnant for approximately 14 days, was deeply anesthetized by isoflurane inhalation and euthanized by cervical spondylolysis. Brains of the fetal rats were excised, placed in ice-cold PBS, and the cerebral cortex was carefully dissected out. The cortices were triturated with a pipette for a few minutes, passed through a 40 μM filter to obtain a single cell suspension, and plated in complete culture media composed of DMEM/F12 (corning,10-092-CV), 1× B27 (Gibco, 17504-044),1× PS (Beyotime, C0222), 20 ng/ml EGF (Peprotech, AF-100-15-100), and 20 ng/ml bFGF (Novus, NBP2-35152). Primary cultures were grown for 4 days and cells were passaged every 3 days. Cells from passages 2 to 4 were used for all experiments.

### Lentivirus transduction

For lentiviral transduction experiments, cells were plated in 24-well plates at a density of 20,000 cells/well. Lentiviral shLbr-1 and shLbr-2, which target CACCAGAGGACCTGTACCTTT and TACCTCCTCTGGTTCTTCCTT, respectively, were transduced into the neural stem cells immediately after passaging, to inhibit the expression of the gene Lbr. Five hundred microliters of the complete culture media was added 12 h later to ensure nutrient-rich growing environment. Seventy-two hours after viral transduction, GFP expression was used to determine the transduction efficiency. Neural stem cells were treated with lentiviral particles at an MOI of 10. Each dish was divided into two halves by marking the cap, and an experimenter blind to the treatment conditions randomly captured one image from each half. The image fields captured covered roughly a 0.96 mm^2^ area, and images were analyzed with ImageJ software (NIH) to quantify the number of neurospheres and morphologically differentiated cells in each image. In each experiment, at least three culture dishes were treated for each condition, and measurements from each of the dishes were averaged. Each experiment was triplicated.

### Western blot

Cultured cells were lysed in a buffer including protease and phosphatase inhibitors. The concentration of total protein was measured by a BCA kit (Beyotime). Protein was denatured at 95 °C for 5 min. Protein (∼30 μg) was loaded for each lane and separated on SDS-PAGE gels (Beyotime). Following the transfer, the blots were incubated overnight at 4 °C with a polyclonal antibody against LBR (1:1000, rabbit; Invitrogen PA5-42709) or a GAPDH antibody (1:20,000, mouse; Sigma G8795) as a control. Following incubation with an horseradish peroxidase–conjugated secondary antibody, the blots were developed by ECL (Pierce). Intensity of the respective bands was analyzed with Image J.

### Immunofluorescence

Cells were grown on coverslips in the wells of 24-well plates. At desired stages, the cells were fixed with 4% paraformaldehyde for 15 min and further permeabilized with 0.3% Triton X-100. Cells were blocked with PBS/3%BSA/1% Triton X-100 and further washed with PBS every 10 min for three times. Cells were incubated with the primary antibody at 4 °C overnight and further washed with PBS every 10 min for three times and followed by an incubation of the secondary antibody at room temperature. The following primary and secondary antibodies were used: mouse anti-GFAP (1:1000, Invitrogen, A-11029), Rabbit anti-NG2 (1:500 MILLIPOREAB5320), Rabbit β-III tubulin (1:500 Abcam ab18207), DAR Cy3 (1:1000 Jakson 712-165-153), DAM Cy3 (1:1000 Jakson 715-165-151).

### Statistical analysis

GraphPad Prism (version 6.01) was used for statistical analysis of the data. For confocal image analysis in either Figure 6 or Figure 7, ten 3 μm Z intervals of consecutive trunk spinal cord sections were stacked with max Z projection in Image J. For analysis in two groups, a two-tailed *t* test (unpaired) was performed. For analysis in three groups, one-way ANOVA was performed. No tests were used to predetermine the sample sizes. Sample sizes were determined by animal availability and the number necessary for definitive results. Data were shown as the mean ± SD. Animal numbers as well as statistical results (*e.g.*, degrees of freedom, test name, and *p* values) were reported in corresponding figure legends. *p* < 0.05 was considered to be statistically significant. One-cell stage embryos were randomized selected for Cas9 or Cas9/sgRNA injections. Live transgenic embryos in the control or experimental group at the desired time points (48 hpf, 72 hpf, or 96 hpf) were all included for confocal analysis. No outliers or samples were excluded from our experiments. For quantification, data were collected from two biological replicates.

## Data availability

The data for this study are available in the Sequence Read Archive of the National Institutes of Health (GEO accession: GSE179096 and GSE186163) and the interactive website https://nantongneurokeylab.shinyapps.io/cell_browser/. Computer codes are available at: https://github.com/lingf85/scRNA-zebrafish.

## Supporting information

This article contains supporting information.

## Conflict of interest

The authors declare no competing interests.
