# Supplementary material for: Expression of myelin transcription factor 1 and lamin B receptor mediate neural progenitor fate transition in the zebrafish spinal cord pMN domain
Source: J Biol Chem. 2022 Sep 5;298(10):102452. doi: 10.1016/j.jbc.2022.102452 (PMC9530849; doi:10.1016/j.jbc.2022.102452)
Supplement: Supplemental Figures S1–S9 and Table S1 [file mmc6.docx]

**Expression of myelin transcription factor 1 and lamin B receptor mediate neural progenitor fate transition in the zebrafish spinal cord pMN domain**

**Lingyan Xing^1#*^, Rui Chai^1#^, Jiaqi Wang^2#^, Jiaqi Lin^2^, Hanyang Li^1^, Yueqi Wang^3^, Biqin Lai^4^, Junjie Sun^1^, Gang Chen^1,5*^**

^1^ Key Laboratory of Neuroregeneration of Jiangsu and the Ministry of Education, Co-innovation Center of Neuroregeneration, NMPA Key Laboratory for Research and Evaluation of Tissue Engineering Technology Products，Nantong University, Nantong, China

^2^ Department of Physiology, School of Medicine, Nantong University, Nantong, China

^3^ School of Medicine, University of Utah, Salt Lake City, USA

^4^ Key Laboratory for Stem Cells and Tissue Engineering (Sun Yat-sen University)，Ministry of Education, Co-innovation Center of Neuroregeneration Nantong University Nantong 226001 China

^5^ Basic Medical Research Center, School of Medicine, Nantong University, Nantong, China

# These authors contributed equally

***Correspondence:** Lingyan Xing and Gang Chen

**Email:** [xlyan011@163.com,](mailto:xlyan011@163.com,) [chengang6626@ntu.edu.cn](mailto:chengang6626@ntu.edu.cn)


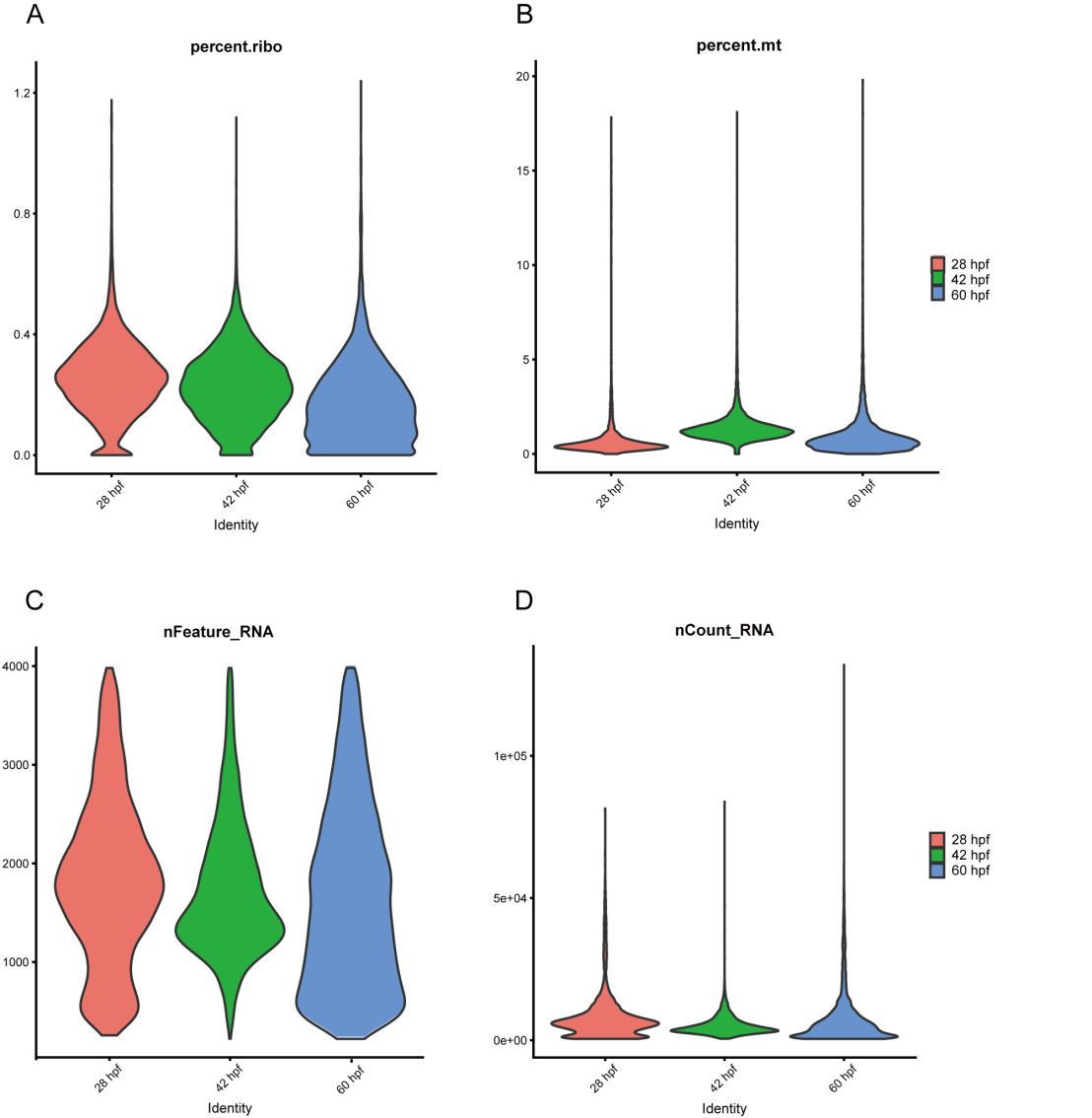


Supplemental Figure 1. Technical performance of scRNA-seq. A-D) The violin plots showing the percentage of ribosome (A) and mitochondrial genes (B), nfeature RNA (the number of genes detected) (C), and RNA counts (UMI) (D).


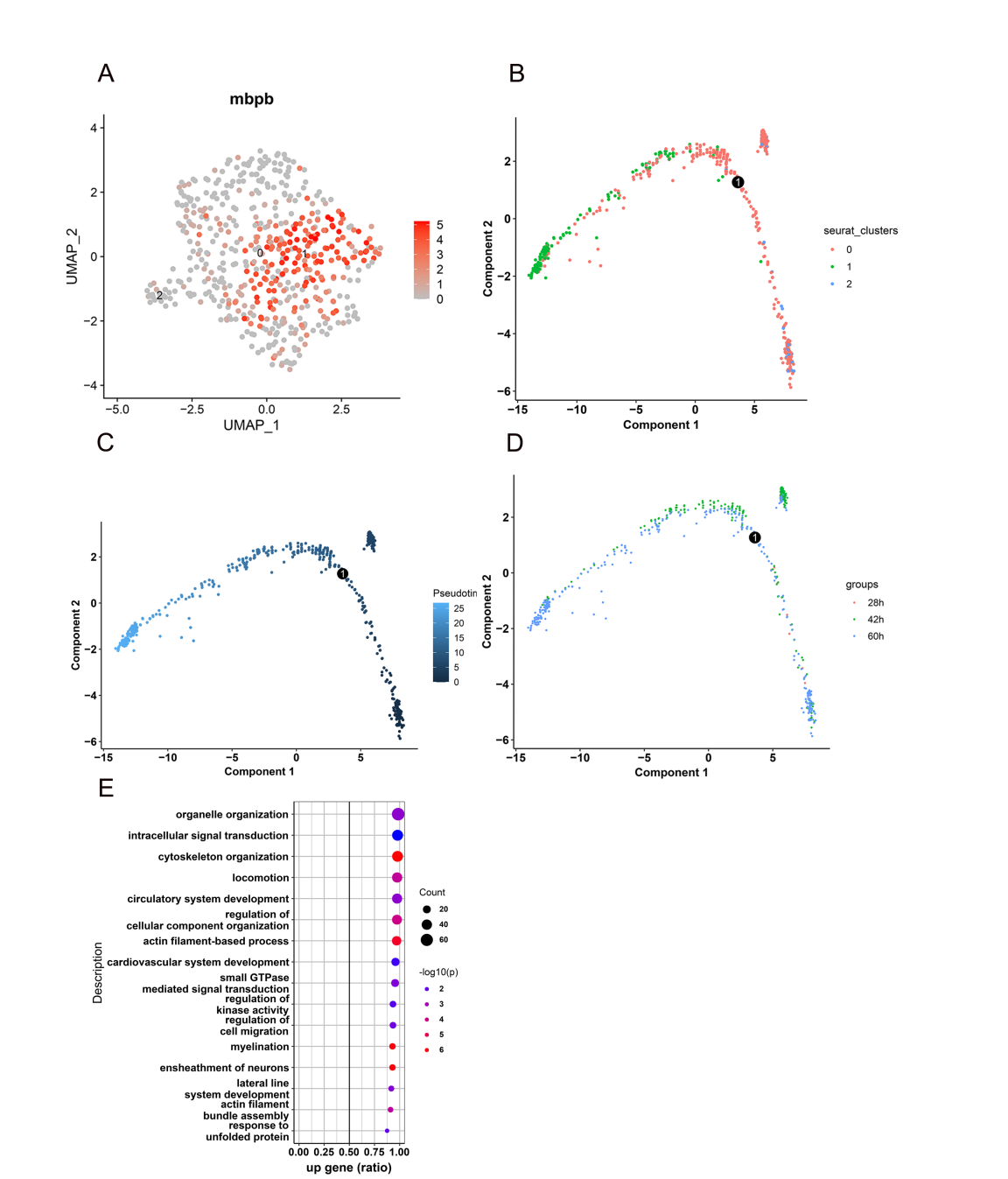


Supplemental Figure 2. Subpopulation and lineage analysis in the OPC/OL cluster. A) UMAP visualization of *mbpb* expression. *Mbpb* is enriched in Subpopulation 1 of OPC/OL. B-C) Pseudotemporal ordering of cells by PAGA. The lighter color depicts more mature cells, and the darker color denotes younger cells (C). D) Pseudotemporal ordering of cells at different time points. E) Go term enriched in the Subpopulation 1.


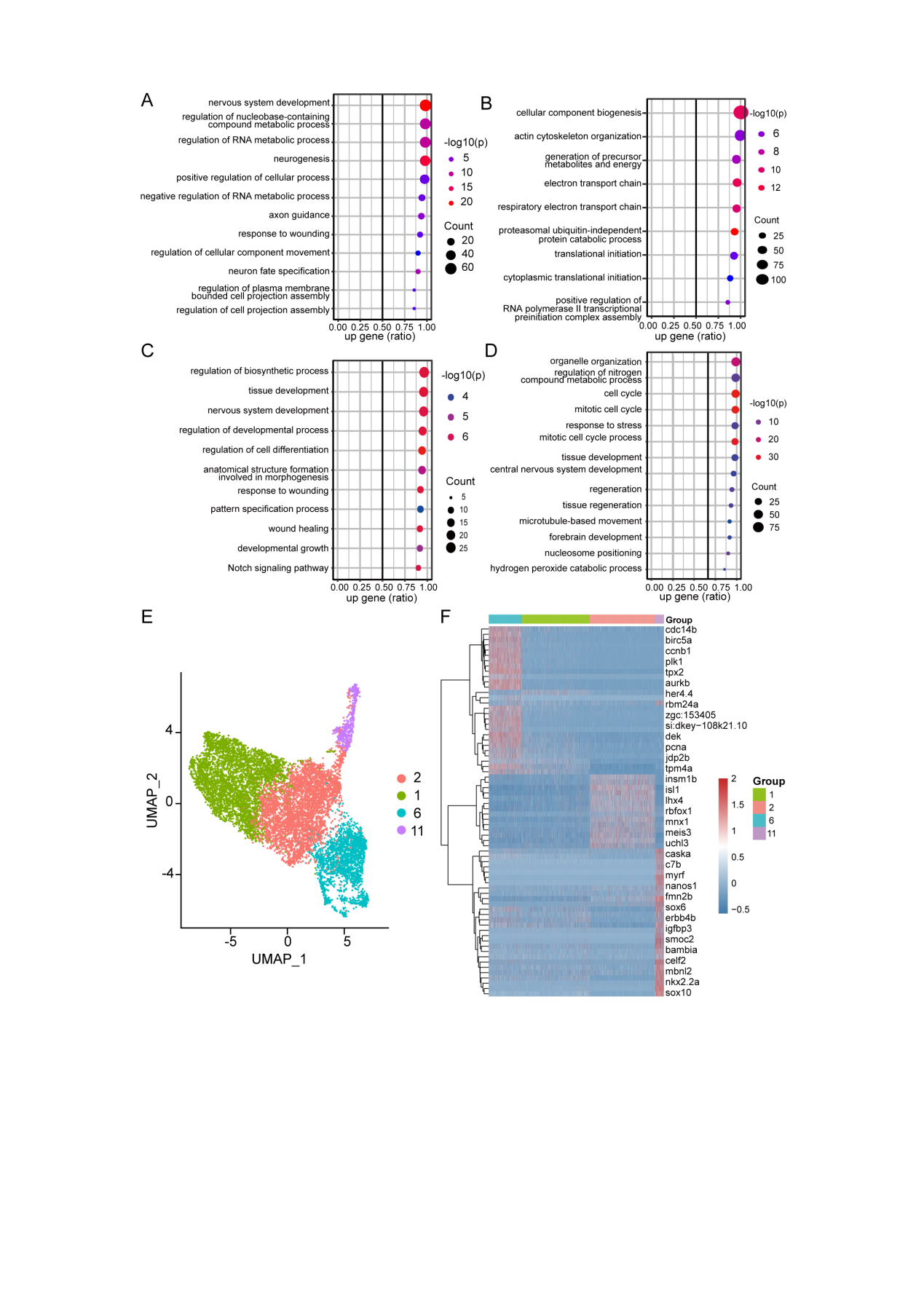


Supplemental Figure 3. Go term enrichment and metabolic genes analysis in progenitors or precursors for motor neurons or OPCs. Go term enrichment in pri-MN (A), OPCs (B), pMN_1 (C), and pMN_2 (D). E) Cell clustering by metabolic genes. The cell clusters shown here are the same as those in Figure 1B. 1, pMN_1; 2, pri-MN; 6, pMN2; 11, OPC/OL. F) Heatmaps of selected metabolic genes in *olig2^+^* progenitors or precursors.


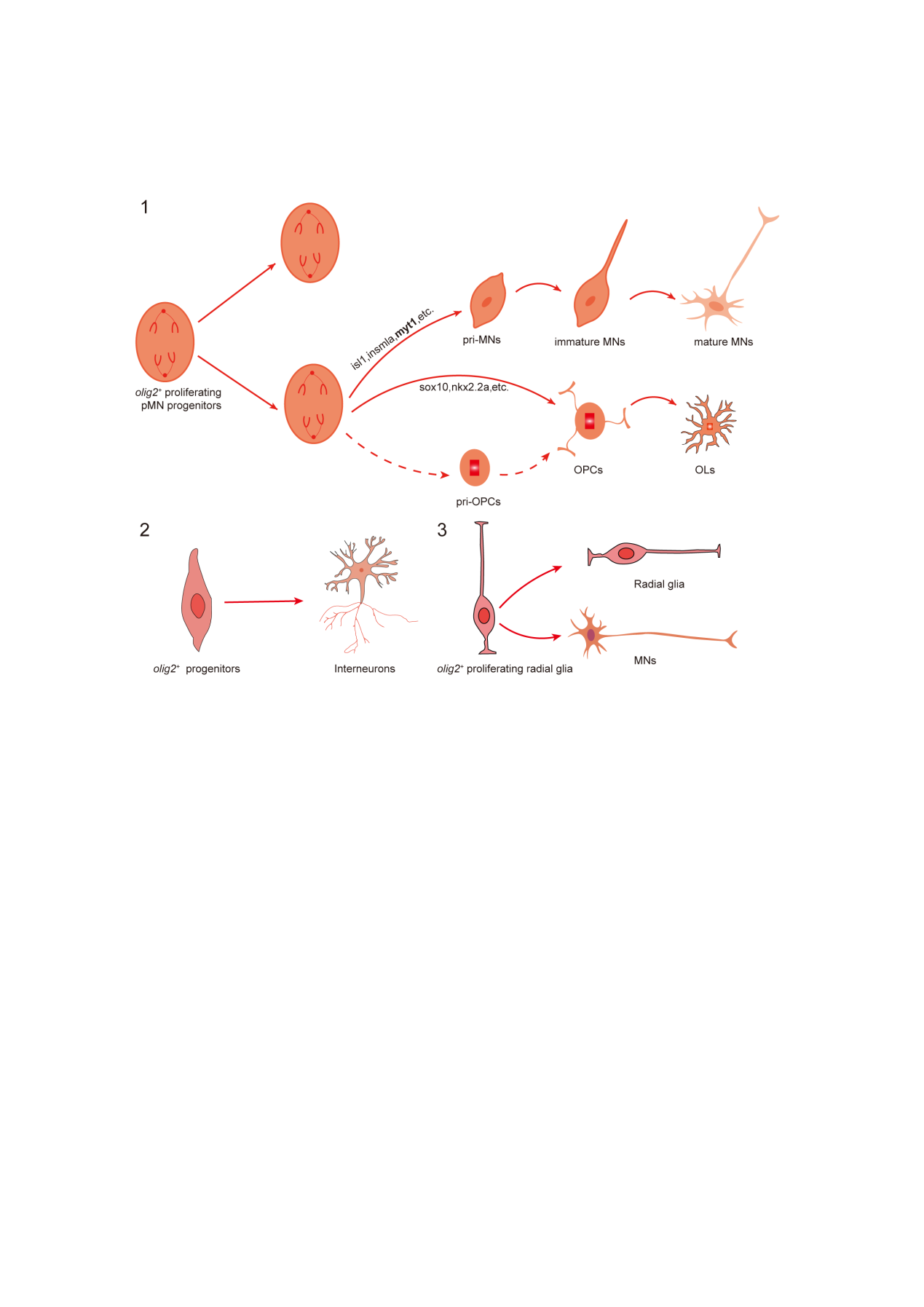


Supplemental Figure 4. Working models for the heterogeneity of *olig2^+^* neural progenitors. *Olig2^+^* progenitors could be pMN progenitors for OPCs and MNs, interneuron progenitors, and radial glia.


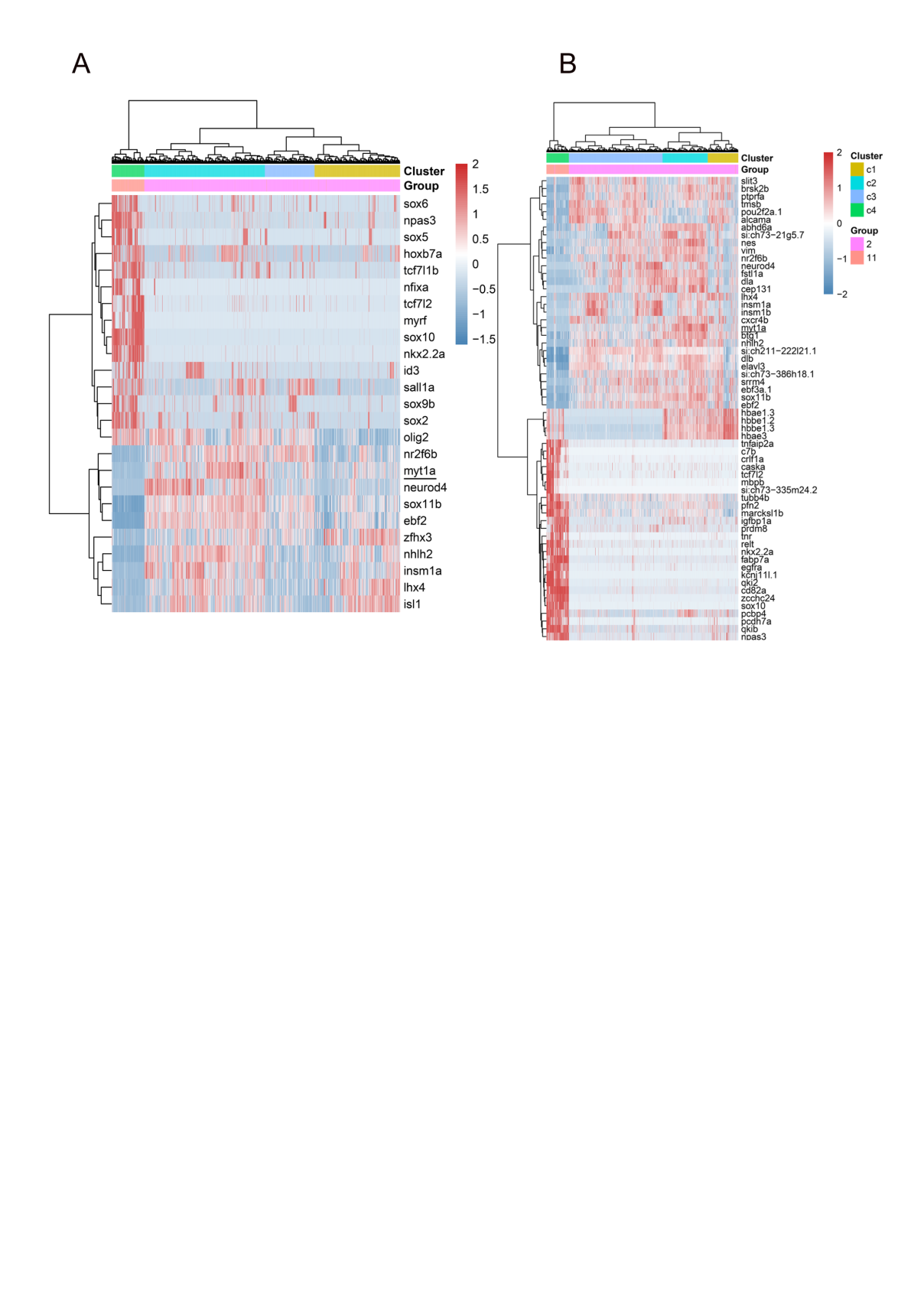


Supplemental Figure 5. Heatmaps of differentially expressed genes or transcription factors in the pri-MN and OPC. A) Differentially expressed transcription factors. B) Top 60 differentially expressed genes. Genes verified *myt1a* are highlighted. Group 2 and 11 are the same as in Figure 1B. 2, pri-MN; OPC/OL. C1-C4 refers to the subclusters of group 2 and 11 with different gene expression patterns.


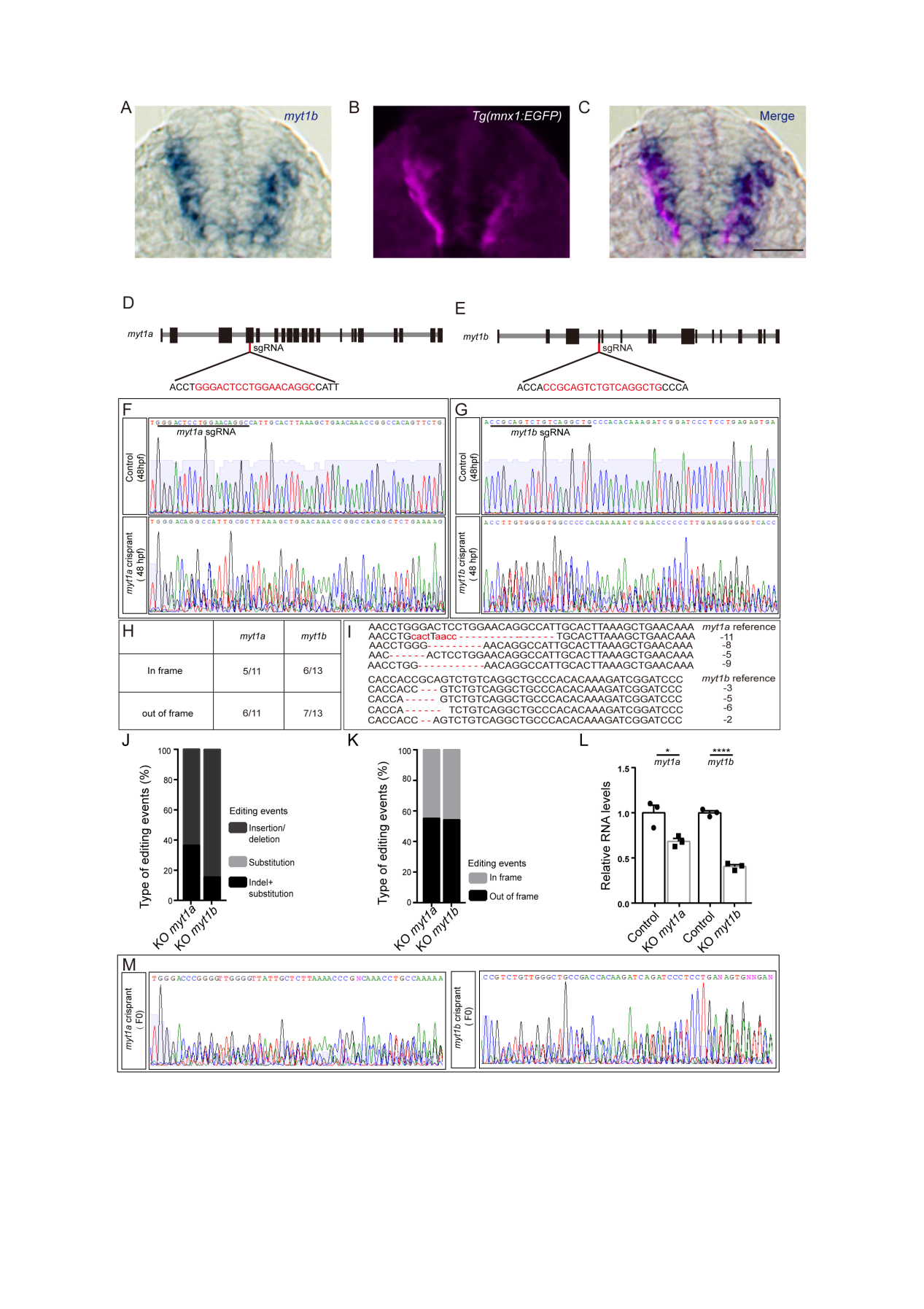


Supplemental Figure 6. *myt1* including *myt1a* and *myt1b* mutagenesis generated by CRISPR. A-C) Transverse sections of spinal cord showing *myt1b* RNA (blue) in *Tg(mnx1:EGFP)* (pink) embryos. Dorsal up. Scale bar, 25 μm. D-E) Schematic of *myt1a* and *myt1b* sgRNAs used for CRISPR. F-G) Representative Sanger sequencing results of *myt1a* or *myt1b* somatic mutagenesis at 48 hpf. H-I) Gene editing types from individual *myt1* or *myt1b* PCR amplicons. J-K) Types of *myt1a* or *myt1b* somatic mutagenesis generated by CRISPR. L) qPCR showing a reduction of *myt1a* and *myt1b* in their respective crispant. M) Representative Sanger sequencing results of *myt1* or *myt1b* somatic mutagenesis in adult F0.


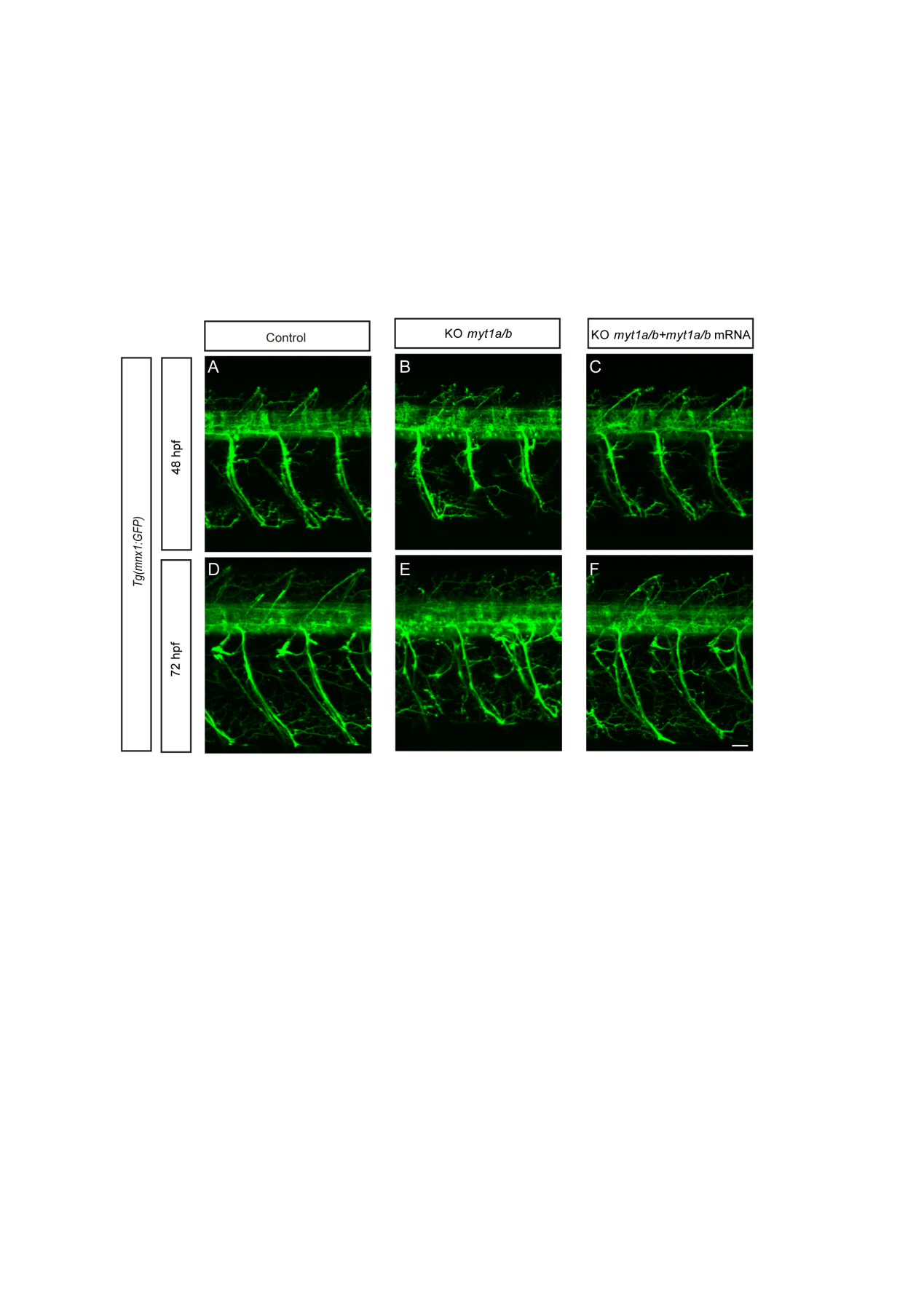


Supplemental Figure 7. Enlarged confocal images of primary motor neurons in control, KO *myt1a/b*, and mRNA rescue groups at 48 hpf (A-C) and 72 hpf (E-F) *Tg(mnx1:GFP)*. These enlarged images are from the Figure 6D-I. Scale bar = 50 μm.


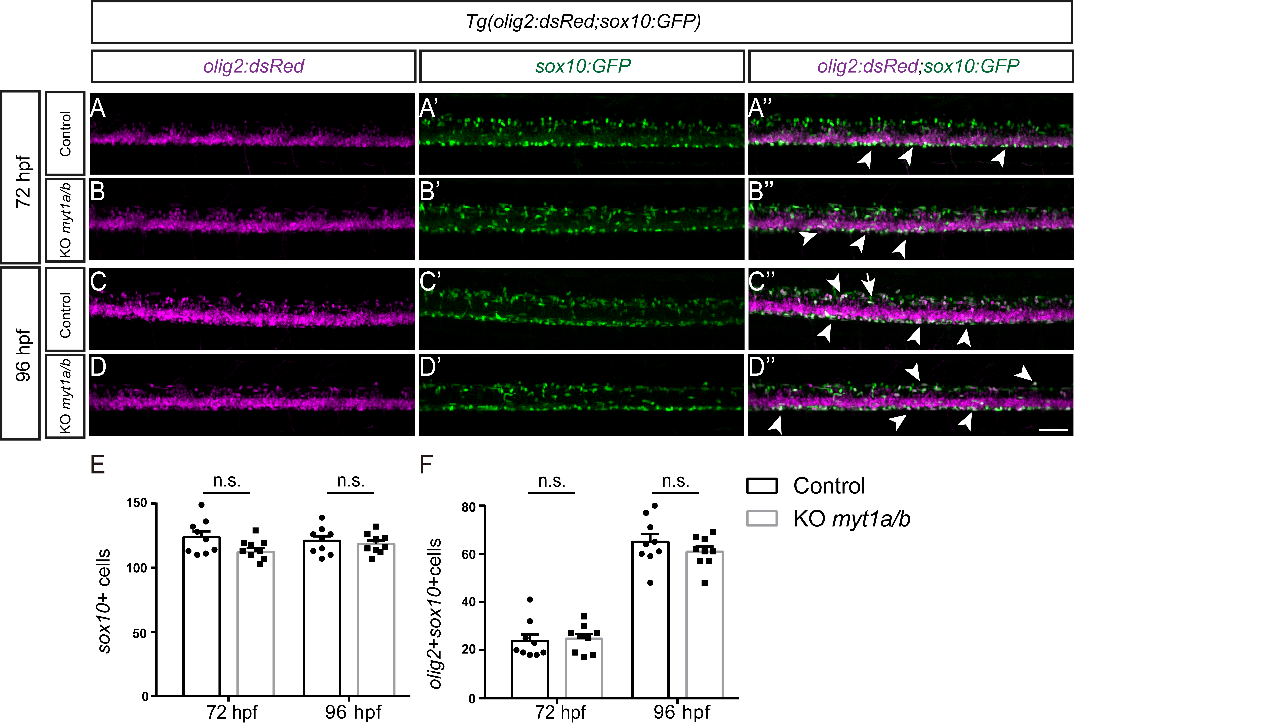


Supplemental Figure 8. *myt1* mutagenesis does not affect OPC specification.

A-D’’) The OPC lineage observed in *Tg(sox10:GFP;olig2:dsRed) at* 72 hpf *and* 96 hpf. Scale bar=25 μm. E) Quantification of *sox10*^+^ cells in controls and KO *myt1a/b* group (n=9 in each group, n.s., no significant). F) Quantification of *sox10*^+^*olig2*^+^ cells in controls and KO *myt1a/b* (n=9 in each group, n.s., no significant).


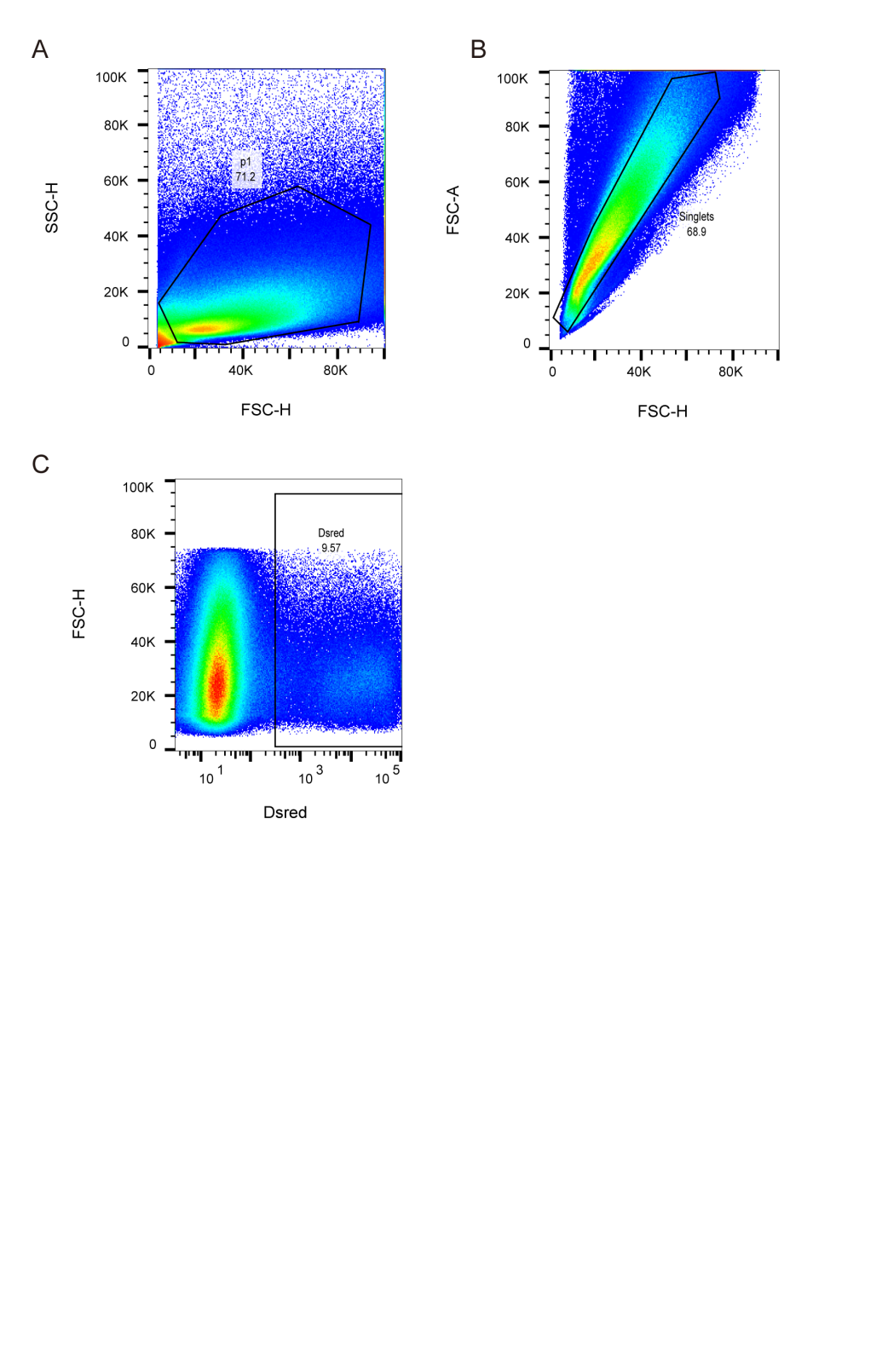


Supplemental Figure 9. FACS gating scatter plots with gating strategy for sorting.

**Supplemental Table 1**

| Zebrafish gene | Forward | Reverse |
| --- | --- | --- |
| *mnx1* | GCGCTCTCGTACTCATACCC | CATGCGCCTGTTCTGAAACC |
| *myt1a* | GAGACCCGAAAACCTCAGCA | ATTCCTCCCTTCTTGGCACG |
| *myt1b* | GGTCTTGTGACCGTGGCATA | GTTGGCGTGTCCCACTAGAA |
| *insm1a* | CGTTCCGGATTGGATGGGAT | AACCCTTGTCAGCAGGTTGT |
| *tac1* | GCGCGCATTGAAGAACTGT | TGATGAGGTCGGGGTTTCCT |

Supplemental Table1: Primers for *in situ* hybridization.

Supplemental Data 1: Significant enriched genes in each cell cluster.

Supplemental Data 2: Enriched Go terms and associated genes in each cell cluster.

Supplemental Data 3: Metabolic pathways and associated genes in the pMN progenitors or precursors.

Supplemental Data 4: Enriched genes in subpopulations of radial glia.

Supplemental Data 5: The top 500 differentially expressed genes (DEGs) that biased the fates of pMN progenitors by Monocle analysis. Transcription factors are marked.
